# From blood to lung tissue: effect of cigarette smoke on DNA methylation and lung function

**DOI:** 10.1186/s12931-018-0904-y

**Published:** 2018-11-03

**Authors:** Maaike de Vries, Diana A van der Plaat, Ivana Nedeljkovic, Rikst Nynke Verkaik-Schakel, Wierd Kooistra, Najaf Amin, Cornelia M van Duijn, Corry-Anke Brandsma, Cleo C van Diemen, Judith M Vonk, H Marike Boezen

**Affiliations:** 1 University of Groningen, University Medical Center Groningen, Department of Epidemiology, Hanzeplein 1, Groningen, 9713 GZ The Netherlands; 2 University of Groningen, University Medical Center Groningen, Groningen Research Institute for Asthma and COPD (GRIAC), Groningen, The Netherlands; 3 Department of Epidemiology, Erasmus Medical Center, Rotterdam, The Netherlands; 4 University of Groningen, University Medical Center Groningen, Department of Obstetrics and Gynaecology, Groningen, The Netherlands; 5 University of Groningen, University Medical Center Groningen, Department of Pathology and Medical Biology, Groningen, The Netherlands; 6 University of Groningen, University Medical Center Groningen, Department of Genetics, Groningen, The Netherlands

**Keywords:** Cigarette smoking, Lung function, DNA methylation, EWAS, Lung tissue

## Abstract

**Background:**

Genetic and environmental factors play a role in the development of COPD. The epigenome, and more specifically DNA methylation, is recognized as important link between these factors. We postulate that DNA methylation is one of the routes by which cigarette smoke influences the development of COPD. In this study, we aim to identify CpG-sites that are associated with cigarette smoke exposure and lung function levels in whole blood and validate these CpG-sites in lung tissue.

**Methods:**

The association between pack years and DNA methylation was studied genome-wide in 658 current smokers with >5 pack years using robust linear regression analysis. Using mediation analysis, we subsequently selected the CpG-sites that were also associated with lung function levels. Significant CpG-sites were validated in lung tissue with pyrosequencing and expression quantitative trait methylation (eQTM) analysis was performed to investigate the association between DNA methylation and gene expression.

**Results:**

15 CpG-sites were significantly associated with pack years and 10 of these were additionally associated with lung function levels. We validated 5 CpG-sites in lung tissue and found several associations between DNA methylation and gene expression.

**Conclusion:**

This study is the first to validate a panel of CpG-sites that are associated with cigarette smoking and lung function levels in whole blood in the tissue of interest: lung tissue.

**Electronic supplementary material:**

The online version of this article (10.1186/s12931-018-0904-y) contains supplementary material, which is available to authorized users.

## Background

Chronic Obstructive Pulmonary Disease (COPD) is a chronic and progressive inflammatory lung disease with cigarette smoking as the main risk factor. COPD is characterized by persistent airflow limitation and COPD patients suffer from severe respiratory symptoms, overall resulting in a poor quality of life [1]. The global prevalence of COPD is 10.7% [2], resulting in a high economic and societal burden. In 2015, 5% of all deaths globally were caused by COPD and it is expected that COPD will be the third leading cause of death worldwide in 2030 (WHO 2016).

The development of COPD is known to be associated with both genetic [3–5] and environmental factors [6] and their interactions [7]. However, the genome-wide association studies (GWAS) and genome-wide interaction studies (GWIS) that have been performed so far identified variants in COPD susceptibility genes that only explain a very small part of the variation in the onset of COPD [8]. As a consequence, the epigenome is increasingly recognized as an important link between changes to the inherited genome and environmental exposures such as cigarette smoke [9]. The epigenome comprises several epigenetic mechanisms that affect gene expression without modifying the DNA sequence. These epigenetic mechanisms are highly dynamic and changes can be induced by environment exposures, diseases and ageing [10].

One well-defined epigenetic mechanism is DNA methylation, which is tissue-specific and involves the binding of a methyl group to a cytosine base adjacent to a guanine base, a so called CpG-site. CpG rich sites are found in the regulatory regions of the DNA and methylation of CpG-sites in these regulatory regions leads to a decrease in gene expression [11]. It has been shown that DNA methylation is highly affected by environmental exposures such as air pollution and cigarette smoke [12–14]. Next to the fact that exposure to cigarette smoke is an important risk factor for COPD, it is also strongly associated with lower lung function levels [15, 16]. Hence, we postulate that DNA methylation plays an important role in the etiology of COPD by mediating the effect that cigarette smoking has on lung function levels. In this study, we aim to identify these CpG-sites by performing an epigenome-wide association study (EWAS) in whole blood in current smokers and validate these smoking-related differences in DNA methylation in lung tissue.

## Methods

### Study design

**Fig. 1:**
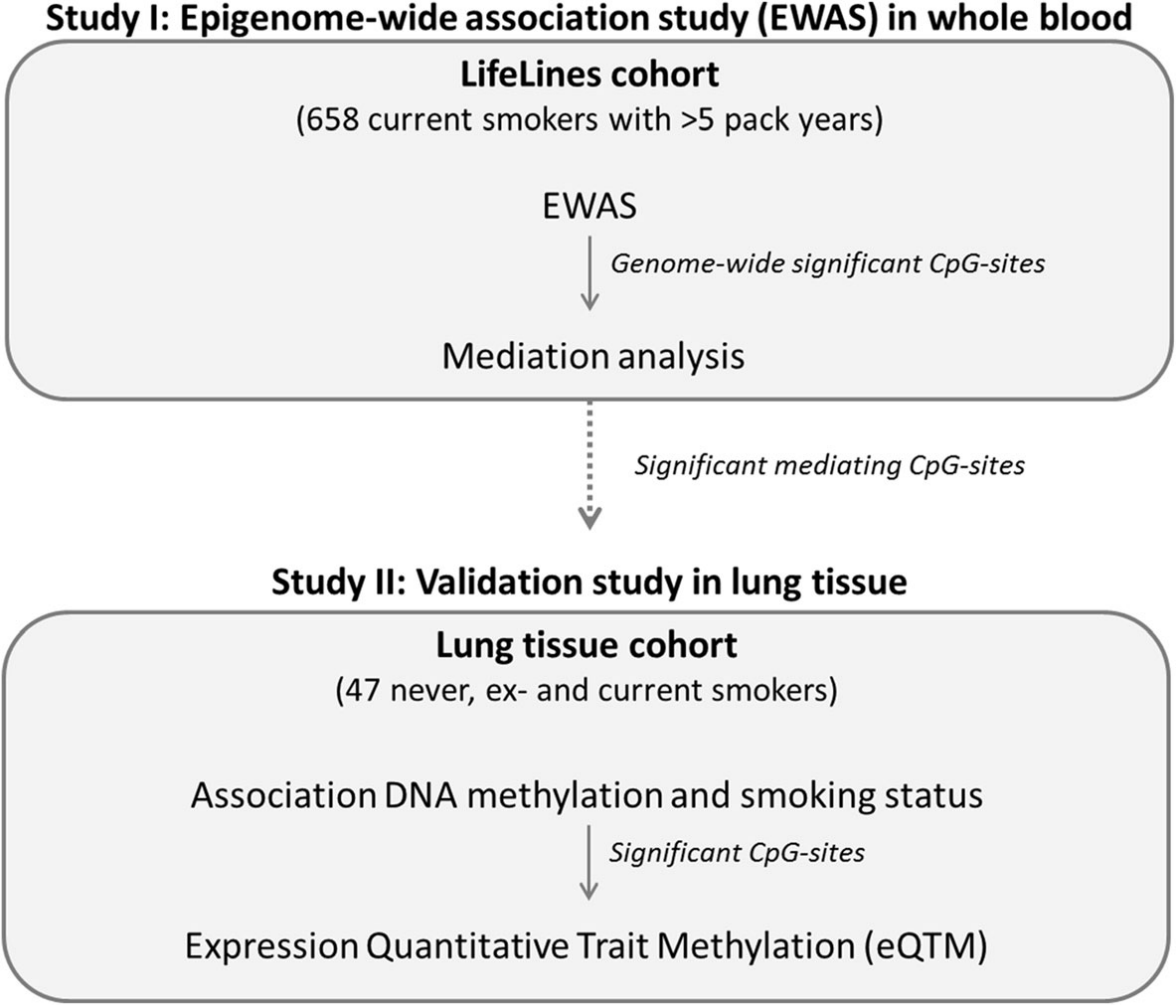
Flowchart of the study

As indicated in the flowchart in Fig. 1, our complete study can be divided into two consecutive studies.

#### Study I: Epigenome-wide association study (EWAS) in whole blood

We performed an epigenome-wide association study (EWAS) in whole blood to identify differential DNA methylation related to pack years in a selection of current smokers from the LifeLines population-based cohort study. Subsequently, we studied whether the identified CpG-sites were also associated with lung function levels using mediation analysis.

#### Study II: Validation study in lung tissue

We validated the significant mediating CpG-sites in actual lung tissue. Subsequently, we studied the association between these CpG-sites and gene expression in lung tissue.

A detailed description of the study population and measurements can be found in the online supplement (Additional file 1).

### Study I: Epigenome-wide association study (EWAS) in whole blood

#### Study population

The association between pack years as a cumulative measurement of cigarette smoking and DNA methylation in whole blood was studied in 658 subjects from the Dutch general population-based cohort study LifeLines at baseline [17]. All selected subjects were current smokers with a smoking history of at least 5 pack years.

#### Measurements

DNA methylation levels in whole blood were determined with the Illumina Infinium Human Methylation 450 K array. After quality control, the final dataset contained DNA methylation data presented as beta values for 420,938 CpG probes [18] (See supplementary methods for sample processing and quality control).

#### Statistical analyses

##### Epigenome-wide association analysis (EWAS) in whole blood

We assessed the association between pack years in current smokers and epigenome-wide DNA methylation in whole blood. We performed robust linear regression using the *MASS* package in R and adjusted the model for the potential confounders age and gender. To adjust for technical variation, principal components were calculated using the 220 control probes incorporated in the Illumina 450 K chip. The principal components that explain >1% of the technical variation (*N* = 7) were included in the analysis. To adjust for the cellular heterogeneity of the whole blood samples, we included proportional white blood cell counts of mononuclear cells, lymphocytes, neutrophils and eosinophils obtained by standard laboratory techniques.

##### Mediation analysis

To assess if the CpG-sites identified in the EWA study were also associated with lung function levels, we performed mediation analysis using the R-package *Mediation* [19]. For every individual CpG-site, the Average Causal Mediation Effect (ACME), Average Direct Effect (ADE), Total Effect and Proportion Mediated was calculated. When the *p*-value of ACME was below 0.05 for a particular CpG-site (tested 2-sided), this CpG-site was considered to be significantly associated with lung function levels.

### Study II: Validation study in lung tissue

#### Study population

DNA methylation in lung tissue was studied in patients undergoing lung resection, lung volume reduction or transplant surgery in the University Medical Center Groningen as previously described [20]. If resection surgery was conducted for tumor removal, macroscopically normal lung tissue was taken far distant form the tumor. All samples were histologically checked for abnormalities using standard haematoxylin and eosin staining. To reach a power of 90% with an alpha of 5%, we selected a total of 47 subjects, being 16 never smokers, 15 ex-smokers and 16 current smokers. The power analysis can be found in the online supplement. All ex- and current smokers had a smoking history of >5 pack years and all ex-smokers quitted smoking for at least 1 year.

We studied gene expression in 36 of the 47 selected subjects included in our lung tissue validation study [20].

#### Measurements

##### DNA methylation in lung tissue

DNA methylation in lung tissue was determined with the PyroMark Q48 Autoprep System. Quality of the pyrosequencing was assessed for every CpG-site in all the subjects individually and the subject was excluded for a particular CpG-site if the default software quality control was not passed. Primers for cg01940273 did not match with the original sequence and cg01940273 was excluded from the validation study. Primer sequences are shown in the online supplement.

##### Gene expression in lung tissue

Total RNA was extracted from lung tissue at Rosetta Inpharmatics Gene Expression Laboratory (Seattle, WA, USA). mRNA profiling was performed using a custom-made Affymetrix HU133 array (GPL 10379) containing 751 controls probe sets and 51,627 non-control probe sets. Gene expression was normalized with the Robust Multichip Average method implemented in the Affymetrix Power Tools software [20].

#### Statistical analysis

##### DNA methylation in lung tissue

The association between smoking status and DNA methylation in lung tissue was studied using linear regression analysis using R. The analyses were adjusted for the potential confounders age and gender.

##### Expression quantitative trait methylation (eQTM) analysis in lung tissue

For the CpG-sites that were differentially methylated in lung tissue, we tested the association between DNA methylation levels and gene expression, i.e. expression quantitative trait methylation (eQTM) analysis. Per CpG-site, all the gene expression probe sets within 1 MB on either side were selected. An association was considered significant when the *p*-value was lower than the Bonferroni adjusted p-value (=0.05 / the number of probe sets within a 2 MB window of the indicated CpG-site).

## Results

### Study I: Epigenome-wide association study (EWAS) in whole blood

#### Study population

Population characteristics are shown in Table 1. Notably, the study population was non-random, i.e. selected based on the presence or absence of airway obstruction.

**Table 1:** Population characteristics

	LifeLines Cohort
Number	658
Males, N (%)	375 (57,0)
Age, years (range)	46 (22–79)
Pack year, years, mean (range)	20,6 (5–100)
FEV_1_/FVC (%), mean (range)	71,7 (40,1 – 92,4)
FEV_1_(%pred)*, mean (range)	94,4 (47,7 – 138,4)
COPD cases, N (%)	279 (42,4)
GOLD COPD Stage ≥ 2†, N (%)	102 (15,6)

* Calculated with GLI-2012 if possible

† COPD GOLD stage > 2 (FEV_1_/FVC < 70% and FEV_1_ between 50 and 80% of predicted)

COPD, Chronic Obstructive Pulmonary Disease; FEV_1_, Forced Expiratory Volume in 1 s; FVC, Forced Vital Capacity

#### Epigenome-wide association analysis (EWAS) in whole blood

**Fig. 2:**
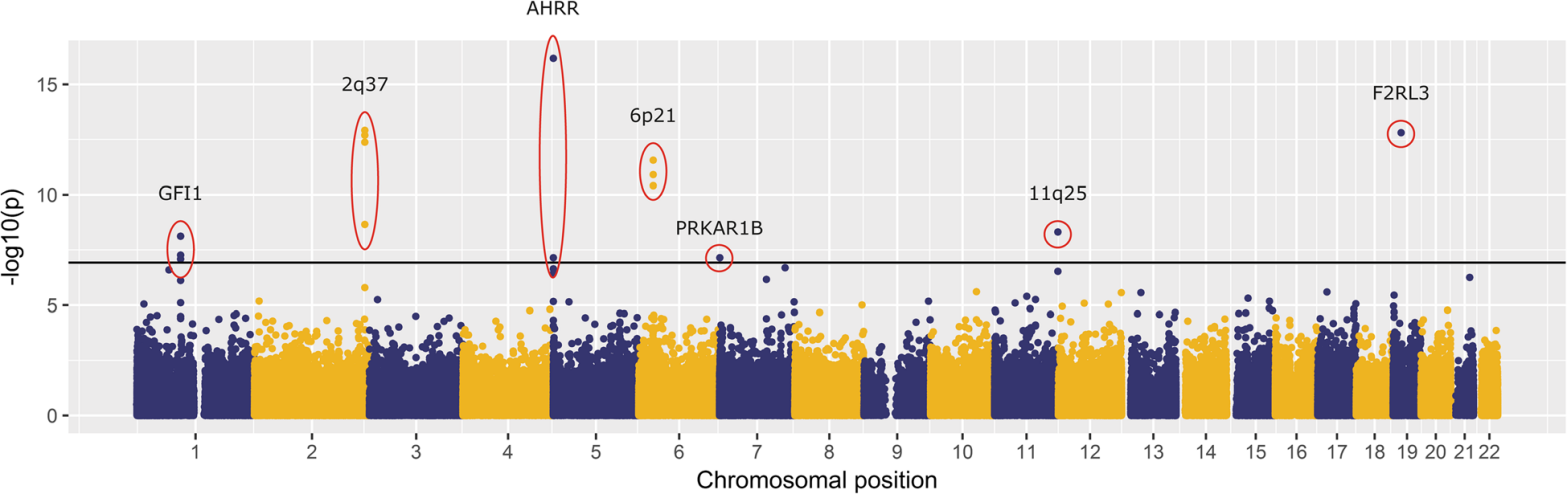
*Manhattan plot of the EWAS on pack years in current smokers.* Every dot represents an individual CpG-site. Location on the X-axis indicates the chromosomal position and location on the Y-axis indicates the inversed log [10] *p*-value. Horizontal line indicates genome wide significance (*p* < 1.19 × 10^^− 7^)

The EWAS in whole blood identified 15 CpG-sites that were significantly (Bonferroni corrected *p*-value < 0.05/420938 = 1.19 × 10^^− 7^) associated with pack years (Fig. 2). Higher number of pack years was associated with lower levels of DNA methylation in 13 of the 15 identified CpG-sites (see Table 2). We identified two novel CpG-sites, cg22994830 annotated to Protein Kinase CAMP-Dependent Type 1 Regulatory Subunit Beta *(PRKAR1B)* and cg20451986 located in the intergenic region of *11q25* between the genes *loc100126239* and Junctional Adhesion Molecule 3 (*JAM3*). As indicated in Table 2, we identified several CpG-sites to be related to pack years, that were described in earlier studies in association with smoking status [12, 21–24]. An overview of all significant CpG sites at nominal *p*-value < 0.05 can be obtained upon request by the corresponding author.

**Table 2:** Genome-wide significant results of EWAS in whole blood

CpG-site	B	SE	*P*-value	Adj. *P*-value	Gene annotation	Gene function^#^
cg05575921*	− 0.00360	0.00043	6.80E-17	2.86E-11	AHRR	Repressor of AHR, important in dioxin toxicity and involved in regulation of cell growth and differentiation
cg21566642*	− 0.00176	0.00024	1.21E-13	5.09E-08	*NA*	*NA*
cg03636183*	− 0.00211	0.00029	1.55E-13	6.53E-08	F2RL3	Plays a role in platelet activation and hypomethylation may be associated with human lung cancer
cg05951221*	− 0.00130	0.00018	2.01E-13	8.48E-08	*NA*	*NA*
cg01940273*	− 0.00143	0.00020	4.20E-13	1.77E-07	*NA*	*NA*
cg06126421*	− 0.00230	0.00033	2.75E-12	1.16E-06	*NA*	*NA*
cg15342087*	− 0.00092	0.00014	1.23E-11	5.19E-06	*NA*	*NA*
cg24859433*	− 0.00089	0.00014	3.91E-11	1.65E-05	*NA*	*NA*
cg27241845*	− 0.00110	0.00018	2.21E-09	9.30E-04	*NA*	*NA*
cg20451986	0.00104	0.00018	4.85E-09	2.04E-03	*NA*	*NA*
cg12876356*	− 0.00244	0.00042	7.55E-09	3.18E-03	GFI1	Transcription repressor essential for hematopoiesis
cg09935388*	− 0.00218	0.00040	5.42E-08	2.28E-02	GFI1	Transcription repressor essential for hematopoiesis
cg21161138*	− 0.00106	0.00020	7.10E-08	2.99E-02	AHRR	Repressor of AHR, important in dioxin toxicity and involved in regulation of cell growth and differentiation
cg22994830	0.00102	0.00019	7.17E-08	3.02E-02	PRKAR1B	Involved in cAMP signaling in cells
cg18146737*	− 0.00256	0.00048	8.19E-08	3.45E-02	GFI1	Transcription repressor essential for hematopoiesis

*Associated with smoking status in previous studies

^#^Gene function obtained by www.genecards.org

#### Mediation analysis

The mediation analysis revealed that 10 CpG-sites were significantly associated with lung function levels (Additional file 2).

### Study II: Validation study in lung tissue

#### Study population

We validated DNA methylation levels in lung tissue of 47 subjects, divided into 3 equal groups based on smoking status (see Additional file 3 for subject characteristics).

#### DNA methylation in lung tissue

**Fig. 3:**
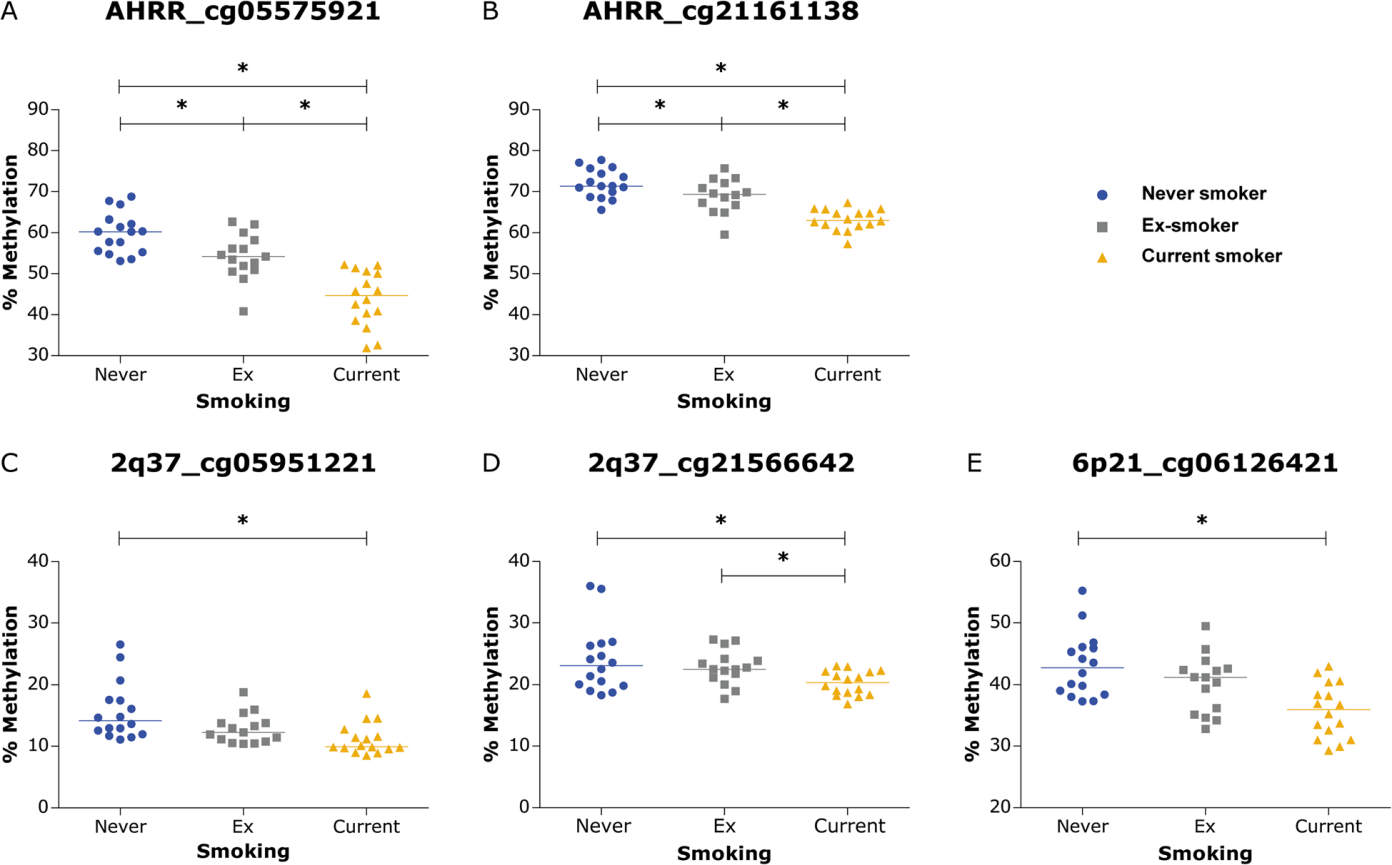
*DNA methylation levels in lung tissue.* CpG-sites that differ in DNA methylation levels between never smokers (blue circles), ex-smokers (gray squares) and current smokers (yellow triangles) are shown

Figure 3 shows that the current smokers had significant lower DNA methylation levels at 4 out of the 9 CpG sites than never smokers (Bonferroni corrected p-value of 0.05/9=0.00556). For cg21566642, the DNA methylation levels were significantly different between current and never smokers at a nominal p-value of 0.05. An overview of the complete analysis can be found in the online supplement (Additional file 4).

#### Expression quantitative trait methylation (eQTM) analysis in lung tissue

**Fig. 4:**
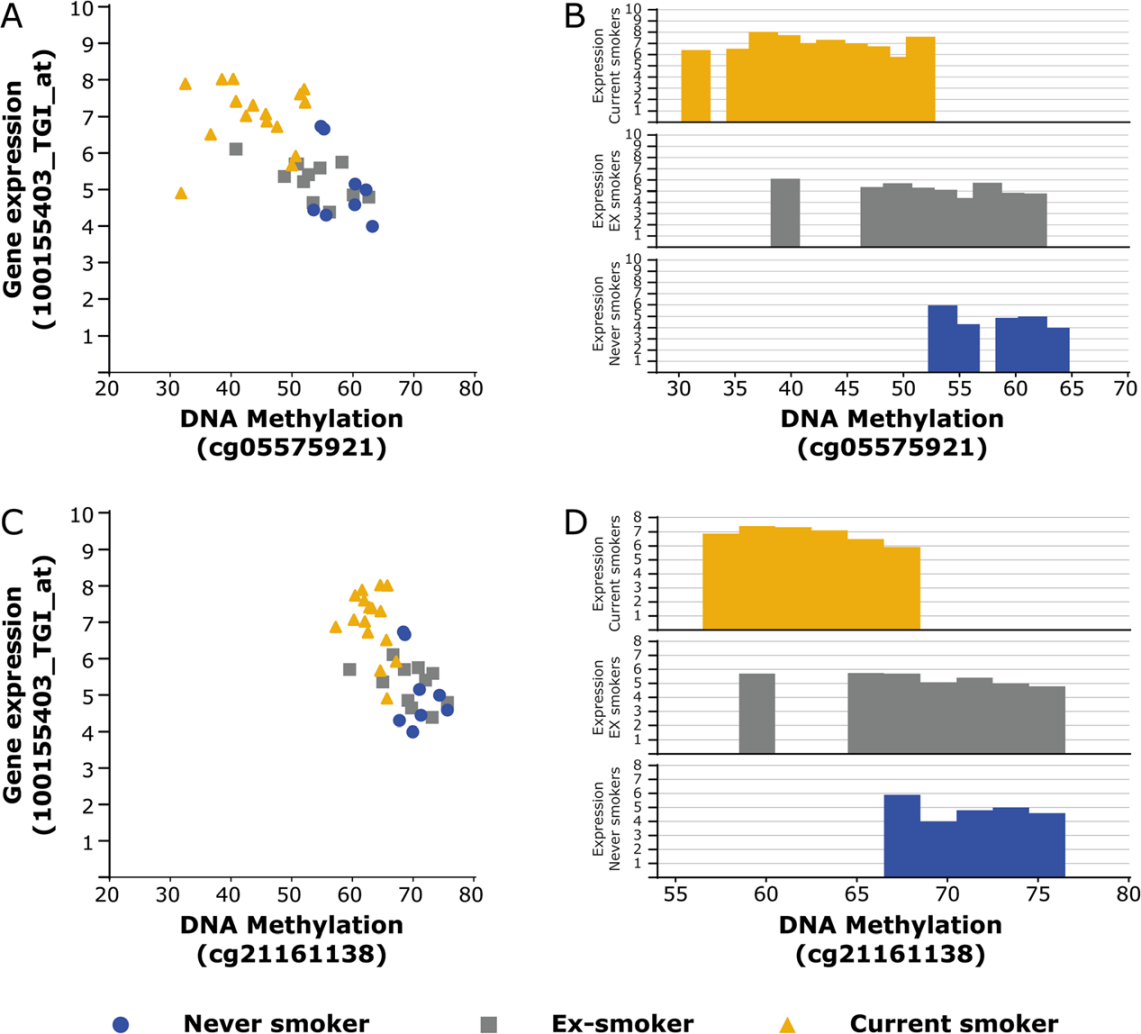
*Association between DNA methylation levels and gene expression of AHRR in lung tissue.* **a** and **c**) DNA methylation levels are presented on the X-axis and the normalized gene expression levels are shown on the Y-axis. Blue circles = never smokers, gray squares = ex-smokers and yellow triangles = current smoker. **b** and **d**) DNA methylation levels are presented on the X-axis and the average of the normalized gene expression levels distributed per 2% DNA methylation are shown on the Y-axis. The upper part with yellow bars represents current smokers, the middle part with gray bars represents ex-smokers and the lower part with blue bars represent never smokers

**Fig. 5:**
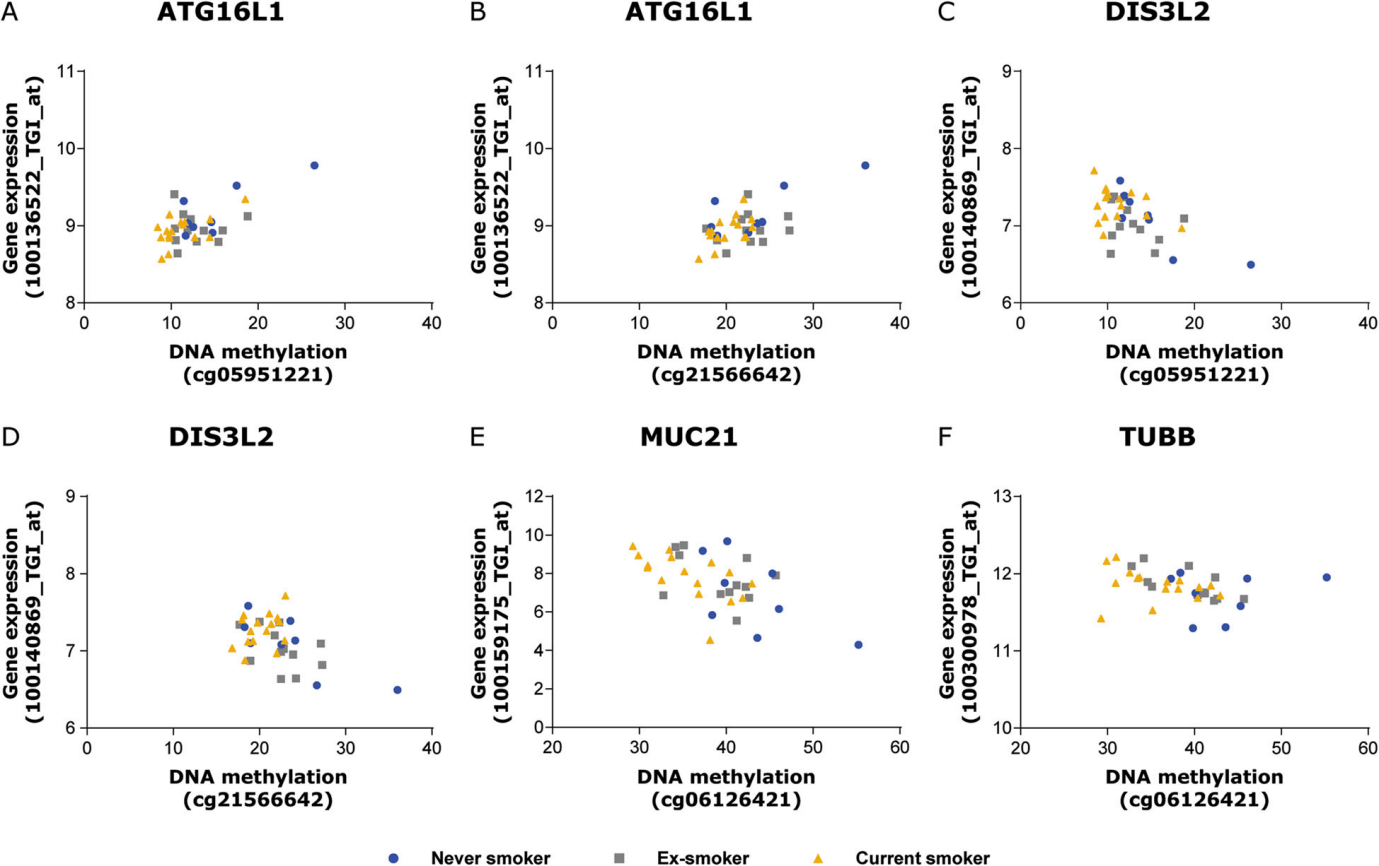
*eQTM analysis for CpG-sites at the intergenic regions on chromosome 2 and 6.* DNA methylation levels are presented on the X-axis en the normalized gene expression levels are shown on the Y-axis. Blue circles = never smokers, gray squares = ex-smokers and yellow triangles = current smokers

DNA methylation at cg05575921 and cg21161138 (the two CpG-sites annotated to *AHRR)* was significantly associated with gene expression levels of *AHRR* in lung tissue (Fig. 4a and c). As indicated in Fig. 4b and d, current smokers showed lower DNA methylation levels together with higher expression levels compared to ex- and never smokers. For the other 3 differentially methylated CpG-sites in lung tissue, we found novel significant associations with gene expression levels. DNA methylation levels at cg05951221 and cg21566642, located in the intergenic region on chromosome 2, were associated with gene expression levels of the genes Autophagy Related 16 Like1 (*ATG16L1*) and DIS3 Like 3′-5′Exoribonuclease (*DIS3L2*) (Fig. 5a-d). DNA methylation levels at cg06126421, located in the intergenic region on chromosome 6, were associated with gene expression levels of the genes Mucin 21 (*MUC21*) and Tubulin Beta Class I (*TUBB*) (Fig. 5e-f). A complete overview of the eQTM analysis can be found in Additional file 5.

## Discussion

In this study, we showed that DNA methylation at 15 CpG-sites was significantly associated with pack years. Next to previously described CpG-sites, we identified 2 novel CpG-sites: cg22994830, annotated to *PRKAR1B*, and cg20451986, located in the intergenic region of chromosome 11. 10 CpG-sites were additionally associated with lung function levels and we validated 5 of these CpG-sites in lung tissue. We found several significant associations between DNA methylation and gene expression in lung tissue. Moreover, we found novel associations for the CpG-sites located in the intergenic regions of chromosome 2 and 6 and biological plausible genes in lung tissue.

In our EWA study, we identified 2 novel CpG-sites associated with pack years. One of them, cg22994830, is located in the body of *PRKAR1B*. As a regulatory subunit of cyclic AMP-dependent protein kinase A, this gene is involved in several cellular events including ion transport, metabolism and transcription. *PRKAR1B* has shown to be implicated in neurodegenerative disorders, however, a role for *PRKAR1B* in pulmonary diseases is currently unknown [25]. The other novel CpG-site cg20451986 has not been annotated to a gene yet. The gene that is closest to this CpG-site, at approximately 10,000 base pairs, is *JAM3*, a gene involved in cell-cell adhesion. While we performed an EWA study on the association between DNA methylation and pack years as a cumulative measurement of exposure to cigarette smoke, as reviewed by *Gao* et al, earlier studies investigated differences in DNA methylation levels between never and current smokers [22]. Nevertheless, we identified several CpG-sites that have been described before. Our third most significant CpG-site cg03636183 located in the gene *F2RL3*, was one of the first sites that was found to be associated with smoking status [23]. In addition, a study by *Zeilinger* et al showed associations of CpG-sites located in the genes *AHRR, GFI1, F2RL3* and the unknown intergenic regions at chromosome 2 and 6 with smoking status [12]. Nine of our identified CpG-sites were identical to the CpG-sites discovered by Zeilinger’s study.

A major strength of our study is that we validated our findings in lung tissue, the actual tissue of interest. This is in contrast to previous studies in which the most significant CpG-sites from the EWAS were validated by replication in whole blood of other populations [12, 13, 23]. In our study, DNA methylation at one of the CpG-sites located in the body of *AHRR,* cg21161138, was significantly lower in current smokers compared to never smokers in lung tissue, in line with previous findings by *Shenker* et al [24]. In addition, the association between DNA methylation of cg21161138 and lower gene expression of *AHRR* in lung tissue also confirms findings of that earlier study. However, we were able to validate 4 additional CpG-sites in lung tissue. The fact that we could confirm a total of 5 CpG-sites, identified in whole blood, in lung tissue suggests that changes in DNA methylation in response to cigarette smoking directly occur at the local level in the lung. For cg03636183 located in the body of *F2RL3,* we neither found differences upon smoking in lung tissue nor an association between DNA methylation levels and gene expression. Since the intensities of the nucleotide peaks in the pyrosequence run of cg03636183 were low compared to other assays, despite the use of several different primers sets, we cannot exclude that actual differences in DNA methylation in lung tissue are masked by technical issues.

Several cross-sectional studies strongly suggest that DNA methylation in whole blood can be partially normalized upon smoking cessation [21, 26, 27]. In our study, we found that DNA methylation levels of ex-smokers are between the levels of never and current smokers for the 5 CpG-sites that are differentially methylated upon smoking. Moreover, with the eQTM analysis, we showed that for *AHRR* both DNA methylation and gene expression levels of ex-smokers tends more towards the levels observed for never smokers compared to current smokers. Even though this is cross-sectional data, it implies a reversible character of DNA methylation.

After identification and validation of CpG-sites that are associated with exposure to cigarette smoke in lung tissue, an important next step is to assess the functional relevance of the CpG-sites. For *AHRR,* the function is well studied. Briefly, *AHRR* inhibits the aryl hydrocarbon receptor pathway, which is involved in the removal of harmful environmental chemicals, including cigarette smoke. Cigarette smoking results in decreased DNA methylation and this decreased DNA methylation subsequently increases the expression of *AHRR*. This leads to reduced removal of harmful compounds and thereby thus potentially increasing the damage caused by these compounds [28]. In contrast, the other 3 CpG-sites that are different with smoking status in lung tissue have not yet been annotated to a gene, making it impossible to assess the potential function of DNA methylation at these sites. By performing an eQTM analysis, we tried to identify the genes that are regulated by these CpG-sites. For the 2 CpG-sites at chromosome 2, we found associations with gene expression of *ATG16L1* and *DIS3L2*. *ATG16L1* is an essential component of the autophagy pathway and mutations in this gene have been associated with inflammatory bowel disease. Since proper function of *ATG16L1* is necessary for host-defense responses against micro-organisms and inflammatory responses in Crohn’s disease, this gene might be relevant in the respiratory system as well [29]. *DIS3L2* belongs to the family of exo-ribonucleases, key enzymes involved in the control of messenger RNA stability. *DIS3L2* has been associated with human diseases such as Perlman syndrome and Wilm’s tumor, but a role in pulmonary diseases has not been described [30]. For cg06126421 located at the intergenic region of chromosome 6, we found an association between DNA methylation levels and the expression of the genes *TUBB* and *MUC21*. *TUBB* encodes a beta tubulin protein of the microtubule cytoskeleton and has shown to be involved in microcephaly with structural brain abnormalities in humans. A role for *TUBB* in lung-related pathologies, however, is currently unknown [31]. *MUC21* belongs to group of 22 mucin proteins, the major glycoprotein components of mucus, which forms the protective layer of the epithelial surface. Since overproduction of mucins is associated with common respiratory diseases including COPD, asthma and cystic fibrosis [32], *MUC21* is a biological plausible gene and further investigations into the inverse association between DNA methylation at cg06126421 and the expression of *MUC21* are warranted.

While our study is one of the first studies to validate a large panel of CpG-sites in actual lung tissue, we have to consider some limitations of our study. First, the results of the mediation analysis should be interpreted with caution. In our study, we used mediation analysis to select CpG-sites that were associated with lung function levels rather than implying biological mediation. We used pack years as cumulative measurement of the exposure to cigarette smoke to assess the association between cigarette smoking and DNA methylation. However, the number of pack years is estimated from self-reported information obtained from questionnaires of the LifeLines population-based cohort study. It has been suggested that self-reported estimations of smoking may underestimate the true smoking prevalence, since cigarette smoking is often interpreted as socially undesirable behavior [33]. Moreover, it has been stated that DNA methylation levels at specific CpG-sites in the genes *AHRR* and *F2RL3* are a better estimate for the exposure to cigarette smoke than pack years derived from self-report [34]. Within the mediation analysis, this potential misclassification of exposure to cigarette smoke may lead to overestimation of the mediation effect [35]. Furthermore, with the cross-sectional design of our study, we cannot infer causality from the mediation analysis. A second potential limitation of our study is that most of the lung tissue was obtained from tumor resection surgery. Although all the tissue has been histologically checked for abnormalities, DNA methylation might be affected by the tumor and potential metastases. However, since this holds for all groups under study, we assume that it will not lead to differences in DNA methylation between the groups.

## Conclusions

We identified 15 CpG-sites that were associated with pack years, of which 2 CpG-sites were novel. From the 10 CpG-sites that were also associated with lung function levels, 5 CpG-sites could be validated in lung tissue. Significant associations with expression levels of biological plausible genes suggest a functional role for DNA methylation in lung tissue. Overall, our study showed for the first time that a panel of CpG-sites associated with cigarette smoking and lung function levels identified in whole blood is also associated with exposure to cigarette smoke in the tissue of interest, i.e. the lung. Further research should reveal the functional relevance of these CpG-sites in the lung and their role in the etiology of COPD.

## Additional files

Additional file 1: Online supplement. (DOCX 158 kb)

Additional file 2: Overview of the results of the mediation analysis. (DOCX 23 kb)

Additional file 3: Subject characteristics lung tissue cohort. (DOCX 17 kb)

Additional file 4: Overview results of the association between DNA methylation and smoking status in lung tissue. (DOCX 19 kb)

Additional file 5: Overview results of the eQTM analysis. (PDF 553 kb)
