# Supplementary material for: From blood to lung tissue: effect of cigarette smoke on DNA methylation and lung function
Source: Respir Res. 2018 Nov 3;19:212. doi: 10.1186/s12931-018-0904-y (PMC6215675; doi:10.1186/s12931-018-0904-y)
Supplement: Supplementary file 1 — Online supplement. (DOCX 158 kb) [file 12931_2018_904_MOESM1_ESM.docx]

**FROM BLOOD TO LUNG TISSUE: EFFECT OF CIGARETTE SMOKE ON DNA METHYLATION AND LUNG FUNCTION**

Maaike de Vries (m.de.vries04@umcg.nl)^1,2^, Diana A. van der Plaat (d.a.van.der.plaat@umcg.nl)^1,2^, Ivana Nedeljkovic (i.nedeljkovic@erasmusmc.nl)^3^, Rikst Nynke Verkaik-Schakel (r.n.schakel@umcg.nl)^4^, Wierd Kooistra (w.kooistra@umcg.nl)^2,5^, Najaf Amin (n.amin@erasmusmc.nl)^3^, Cornelia M. van Duijn (c.vanduijn@erasmusmc.nl)^3^, Corry-Anke Brandsma (c.a.brandsma@umcg.nl)^2,5^, Cleo C. van Diemen (c.c.van.diemen@umcg.nl)^6^, Judith M. Vonk (j.m.vonk@umcg.nl)^1,2^ and H. Marike Boezen (h.m.boezen@umcg.nl)^1,2^

**Online Supplement**

**Methods**

**Study I: Epigenome-wide association study (EWAS) in whole blood**

Study population

*LifeLines Groningen*

The LifeLines study is a multi-disciplinary prospective population-based cohort study and biobank examining health and health-related behavior of persons living in the Northern region of the Netherlands using a three-generation design. LifeLines was established as a resource for research on complex interactions between environmental, phenotypic and genomic factors in the development of chronic diseases and healthy ageing. Participants were recruited via general practioners and underwent physical examination, including lung function, ECG and cognition tests and completed extensive questionnaires (1,2). Written informed consent was provided by all subjects and the study was approved by the Medical Ethics Committee of the University Medical Center Groningen.

To study DNA methylation in whole blood, 1,656 subjects were selected based on specific selection criteria including smoking history (never or current smokers, ex-smokers were excluded), airway obstruction (defined as Forced Expiratory Volume in 1 second to Forced Vital Capacity ratio (FEV_1_/FVC) <70%) and occupational related exposures. To optimize the exposure contrast, only current smokers with more than 5 pack years were selected. For the current study, only the current smokers (N=658) were included.

Measurements

*Genome-wide methylation assay*

DNA methylation levels at approximately 485,000 CpG-sites were determined with the Illumina Infinium Human Methylation 450K array (Illumina Inc) (3). The samples from the 1,656 included subjects were randomized across the array based on sex, exposure and airway obstruction. DNA was isolated from whole blood and 500 ng DNA was bisulfite converted using the EZ-96 DNA methylation kit (Zymo research Corporation). The samples were processes according to the Illumina 450K protocol.

Quality control steps were performed using the Minfi package in R and included the removal of samples with >1% of all probes having a detection p-value >0.01 and samples with an incorrect sex or SNP prediction (4). We removed single probes with a detection p-value >0.01, sex chromosome probes, cross-reactive probes, probes measuring SNPs and probes where the CpG itself or the single base exentsion site is a SNP (5). The data was normalized using DASEN implemented in the watermelon package in R (6).

A principal component (PC) analysis was performed using the 220 control probes included on the Illumina 450K chip to estimate possible batch effects (7). These control probes are related to bisulfite conversion, normalization, staining, extension, hybridization, target removal, specificity and non-polymorphic. PCs that explained >1% of the variance were selected and included as covariate in the analyses. In total, 7 PCs were selected that captured 95.5% of the technical variance.

**Study II: Validation study in lung tissue**

Study population

Lung tissue was collected from patients undergoing lung resection, lung volume reduction or transplant surgery in Groningen (The Netherlands) in accordance with local ethical guidelines as previously described (8,9). Complete lung biopsies were stored at -80 °C until further analysis. If resection surgery was conducted for tumour removal, macroscopically normal lung tissue was taken far distant from the tumour. All samples were histologically checked for abnormalities using standard haematoxylin and eosin staining.

To determine the number of subjects needed in each group to have sufficient power to test if DNA methylation levels in lung tissue were significantly different between the groups, we performed a power-analysis on forehand. Based on the mean methylation beta-values of the CpG-sites of smokers and never-smokers from our DNA methylation cohort, we calculated that 13 subjects were needed in each group to reach a power of 90% with an alpha of 5% (Figure 1). For this study, we selected a total of 47 subjects, including 16 never smokers, 15 ex-smokers and 16 current smokers. All ex- and current smokers had a smoking history of at least 5 pack years and all ex-smokers had quitted smoking for at least 1 year.


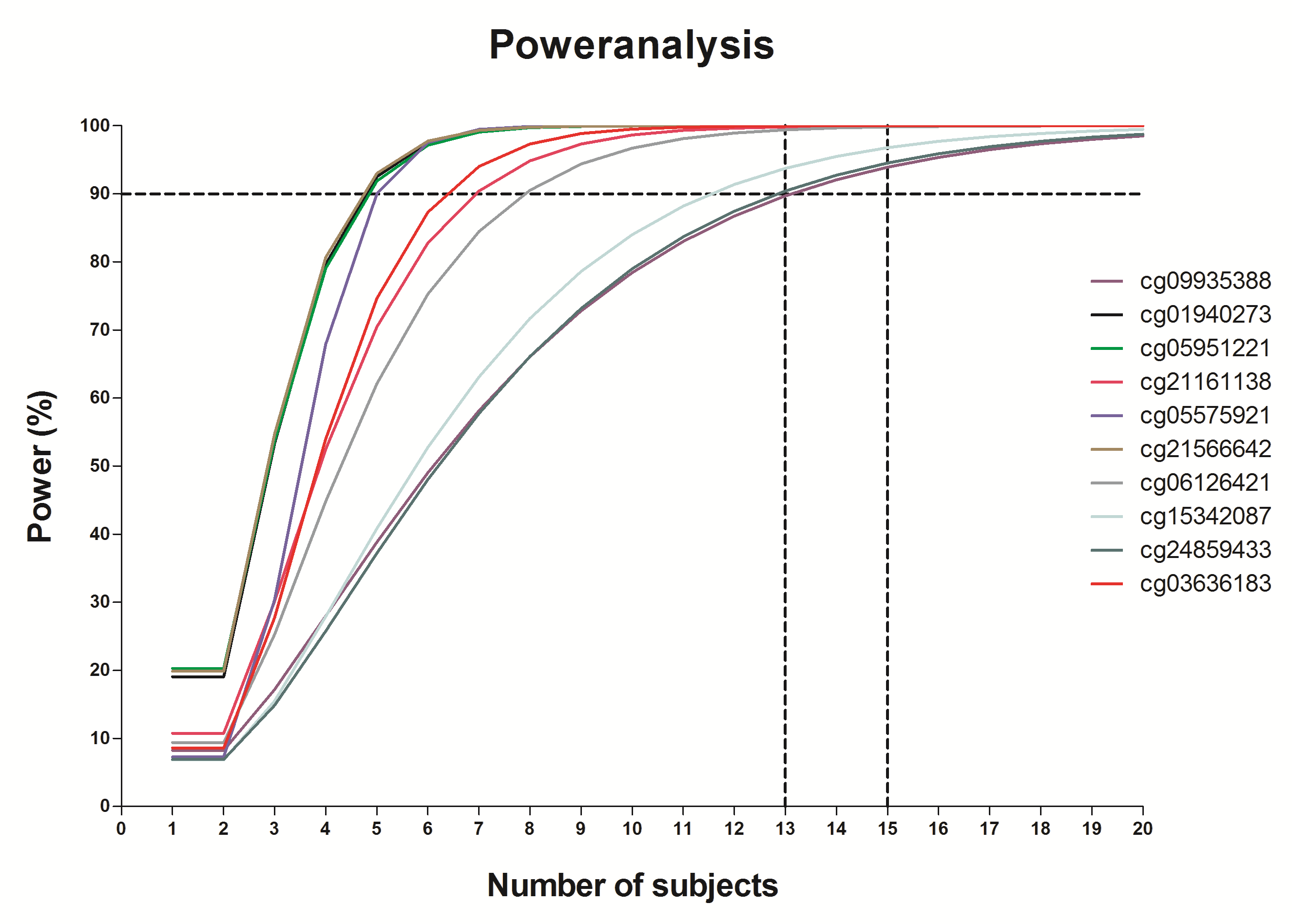


*Figure 1: Estimation of the power for all the selected CpG-sites individually. The power is shown per increase of 1 subject until a total of 20 included subjects.*

Measurement

DNA was isolated from cryosections of frozen lung biopsies using the Allprep DNA/RNA Minikit (Qiagen), thereby homogenizing the samples with a syringe and needle. 500 ng DNA was bisulfite converted using the EZ DNA methylation Gold Kit (Zymo research) conform manufacturer’s instructions. Primers were developed with the Pyromark Assay Design 2.0 (Qiagen) and complete primers sequences are listed in table S5 below. 50 ng of the of bisulfite converted DNA was used as template for each of the subsequent PCRs. DNA was amplified for 45 cycles with the Applied Biosystems Veriti Thermal Cycler (ThermoFisher Scientific) using the HotStartTaq Mastermix (Qiagen). CpG methylation was quantified using the Pyromark Q48 Autoprep System (Qiagen) and the PyroMark Q48 Advanced CpG Reagents (Qiagen). Methylation values were determined with the Pyromark Q48 Autoprep 2.4.1. Software (Qiagen). Quality of the pyrosequencing was assessed for every CpG-site in all the subjects individually and a subject was excluded for a particular CpG-site if the default software quality control was not passed.

*Table S5: Primer sequences Pyrosequencing*

| CpG-sites | Forward primer | Reverse primer | Sequence primer |
| --- | --- | --- | --- |
| cg01940273 | ATTTTATTTGGTTTT  GGTTAGGA * | ATCCTCCTCCCTACACAC | ACCTTCATTACATCT  CT |
| cg03636183 | GGGGTAGAGTTAGGG  TTAGTAGGA | CCAATAACAATAACA  CCCTAAAACTCC * | GTTTATTAGTAGTAT  GGTGGA |
| cg05575921 | GGGGATTGTTTATTT  TTGAGAGGGTAG | ACCTATCCCCTACCT  CCC * | ATTTTTGAGAGGGTA  GT |
| cg05951221 | GTTAGGATGGAGTA  GAGATTAG | CCTTTCTACCCCCC  AAACTTCT * | GGTTTATTTTAAGTA  GGGTTT |
| cg06126421 | TGGTAATTGTTTTGG  AGGGTAGGT | AAAACTCTCTAATAT  TTCTCCTCCTCTA * | TGGAGGGTAGGTTGG |
| cg09935388 | TGGTTATTTTAGTGA  GAGGTTGTAT | AAAACCATAACTCCA  ACAAATATATCTAA * | TTTAGTGAGAGGTTGT  ATT |
| cg21161138 | ATTTTGTAGGGGTT  TTGGTGGT | CAACTCTAACCCCAA  AATCTCT * | GGGTTTTGGTGGTTG |
| cg15342087  cg24859433^#^ | AGGGTTTATTTTTTGG  TAGTTTATTAAAT | CTTCCAAAATTAAAA  AAAACAATTCA * | GGGAGTTGTAGATAG  GT |
| cg21566642 | AGTTGGGGTTTTTGT  ATTTAGGTT | CCTCCTAATAAACCTC  TACATAACC * | TTTTTTTTAGAGGTG  TTTGGGAG |
| cg22994830 | GTTTAGAAGGGAATT  AGTATAGGTTAGG | TTCCTATCTACTTCT  CCCTTTACT * | GGAGTTAATATTGGT  TTTTAGT |
| cg27241845 | TAGTTATAAGTTGTA AGGGGTGATTT | CTCCCCAATTCAAAC AATTCTCCTACC | GTTGTAAGGGGTGAT TTT |

* Biotinylated primer

^#^ The two individual CpG-sites can be determined with the same primers.

**REFERENCES**

(1) Stolk RP, Rosmalen JG, Postma DS, de Boer RA, Navis G, Slaets JP, et al. Universal risk factors for multifactorial diseases: LifeLines: a three-generation population-based study. Eur J Epidemiol 2008;23(1):67-74.

(2) Scholtens S, Smidt N, Swertz MA, Bakker SJ, Dotinga A, Vonk JM, et al. Cohort Profile: LifeLines, a three-generation cohort study and biobank. Int J Epidemiol 2015 Aug;44(4):1172-1180.

(3) van der Plaat DA, de Jong K, de Vries M, van Diemen CC, Nedeljkovic I, Amin N, et al. Occupational exposure to pesticides is associated with differential DNA methylation. Occup Environ Med 2018 Jun;75(6):427-435.

(4) Aryee MJ, Jaffe AE, Corrada-Bravo H, Ladd-Acosta C, Feinberg AP, Hansen KD, et al. Minfi: a flexible and comprehensive Bioconductor package for the analysis of Infinium DNA methylation microarrays. Bioinformatics 2014 May 15;30(10):1363-1369.

(5) Chen YA, Lemire M, Choufani S, Butcher DT, Grafodatskaya D, Zanke BW, et al. Discovery of cross-reactive probes and polymorphic CpGs in the Illumina Infinium HumanMethylation450 microarray. Epigenetics 2013 Feb;8(2):203-209.

(6) Pidsley R, Y Wong CC, Volta M, Lunnon K, Mill J, Schalkwyk LC. A data-driven approach to preprocessing Illumina 450K methylation array data. BMC Genomics 2013 May 1;14:293-2164-14-293.

(7) Lehne B, Drong AW, Loh M, Zhang W, Scott WR, Tan ST, et al. A coherent approach for analysis of the Illumina HumanMethylation450 BeadChip improves data quality and performance in epigenome-wide association studies. Genome Biol 2015 Feb 15;16:37-015-0600-x.

(8) Hao K, Bosse Y, Nickle DC, Pare PD, Postma DS, Laviolette M, et al. Lung eQTLs to Help Reveal the Molecular Underpinnings of Asthma. PLoS Genet 2012 Nov;8(11):e1003029.

(9) Brandsma CA, van den Berge M, Postma DS, Jonker MR, Brouwer S, Pare PD, et al. A large lung gene expression study identifying fibulin-5 as a novel player in tissue repair in COPD. Thorax 2015 Jan;70(1):21-32.
