# Supplementary material for: From blood to lung tissue: effect of cigarette smoke on DNA methylation and lung function
Source: Respir Res. 2018 Nov 3;19:212. doi: 10.1186/s12931-018-0904-y (PMC6215675; doi:10.1186/s12931-018-0904-y)
Supplement: Supplementary file 2 — Overview of the results of the mediation analysis. (DOCX 23 kb) [file 12931_2018_904_MOESM2_ESM.docx]

*Additional file 2: Overview of the results of the mediation analysis*

|  |  | ACME | | | ADE | | | TOTAL EFFECT | | | PROPORTION MEDIATED | | |
| --- | --- | --- | --- | --- | --- | --- | --- | --- | --- | --- | --- | --- | --- |
| CpG-site | **Gene** | **B** | **95% CI** | **P** | **B** | **95% CI** | **P** | **B** | **95% CI** | **P** | **Proportion** | **95% CI** | **P** |
| cg01940273 | 2q37.1 | -0.0344 | [-0.0597;-0.0130] | **0.0000** | -0.0468 | [-0.1159; 0.0184] | 0.1480 | -0.0812 | [-0.1455;-0.0190] | 0.0080 | 0.4238 | [0.1318;1.6747] | 0.0080 |
| cg03636183 | F2RL3 | -0.0306 | [-0.0565;-0.0103] | **0.0020** | -0.0545 | [-0.1186; 0.0058] | 0.0800 | -0.0851 | [-0.1471;-0.0233] | 0.0060 | 0.3591 | [ 0.1084;1.1888] | 0.0080 |
| cg05575921 | AHRR | -0.0459 | [-0.0734;-0.0219] | **0.0000** | -0.0365 | [-0.1046; 0.0271] | 0.2720 | -0.0824 | [-0.1448;-0.0205] | 0.0060 | 0.5574 | [ 0.2240;2.0019] | 0.0060 |
| cg05951221 | 2q37.1 | -0.0371 | [-0.0621;-0.0206] | **0.0000** | -0.0388 | [-0.1040; 0.0295] | 0.2540 | -0.0759 | [-0.1384;-0.0132] | 0.0100 | 0.4893 | [ 0.1992;2.4521] | 0.0100 |
| cg06126421 | 6.p21.33 | -0.0312 | [-0.0548;-0.0137] | **0.0060** | -0.0537 | [-0.1212; 0.0058] | 0.0860 | -0.0849 | [-0.1484;-0.0226] | 0.0060 | 0.3678 | [ 0.1295;1.1416] | 0.0120 |
| cg09935388 | GFI1 | -0.0165 | [-0.0354;-0.0011] | **0.0360** | -0.0700 | [-0.1334;-0.0096] | 0.0180 | -0.0866 | [-0.1490;-0.0238] | 0.0040 | 0.1910 | [ 0.0082;0.6066] | 0.0400 |
| cg12876356 | GFI1 | -0.0104 | [-0.0263; 0.0020] | 0.1060 | -0.0744 | [-0.1383;-0.0134] | 0.0120 | -0.0848 | [-0.1483;-0.0237] | 0.0040 | 0.1223 | [-0.0243;0.4431] | 0.1100 |
| cg15342087 | 6p21.33 | -0.0214 | [-0.0441; 0.0002] | 0.0540 | -0.0614 | [-0.1321; 0.0030] | 0.0600 | -0.0828 | [-0.1467;-0.0212] | 0.0060 | 0.2587 | [-0.0050;1.0632] | 0.0600 |
| cg18146737 | GFI1 | -0.0085 | [-0.0243; 0.0027] | 0.1600 | -0.0760 | [-0.1397;-0.0155] | 0.0100 | -0.0845 | [-0.1480;-0.0234] | 0.0060 | 0.1004 | [-0.0344;0.3769] | 0.1660 |
| cg20451986 | 11q25 | -0.0058 | [-0.0194; 0.0083] | 0.4620 | -0.0773 | [-0.1459;-0.0139] | 0.0120 | -0.0831 | [-0.1474;-0.0226] | 0.0060 | 0.0694 | [-0.1190;0.4088] | 0.4680 |
| cg21161138 | AHRR | -0.0229 | [-0.0422;-0.0080] | **0.0040** | -0.0614 | [-0.1275;-0.0008] | 0.0480 | -0.0843 | [-0.1468;-0.0223] | 0.0060 | 0.2721 | [ 0.0774;0.9306] | 0.0100 |
| cg21566642 | 2q37.1 | -0.0374 | [-0.0630;-0.0166] | **0.0000** | -0.0412 | [-0.1094; 0.0275] | 0.2340 | -0.0787 | [-0.1412;-0.0161] | 0.0080 | 0.4758 | [ 0.1543;2.1505] | 0.0080 |
| cg22994830 | PRKAR1B | -0.0140 | [-0.0298;-0.0001] | **0.0480** | -0.0694 | [-0.1361; -0.0070] | 0.0320 | -0.0835 | [-0.1468;-0.0223] | 0.0040 | 0.1682 | [-0.0007;0.7375] | 0.0520 |
| cg24859433 | 6p21.33 | -0.0301 | [-0.0540;-0.0100] | **0.0060** | -0.0536 | [-0.1213; 0.0063] | 0.0780 | -0.0838 | [-0.1455;-0.0226] | 0.0060 | 0.3598 | [ 0.1004;1.1723] | 0.0120 |
| cg27241845 | 2q37.1 | -0.0134 | [-0.0307; 0.0016] | 0.0900 | -0.0704 | [-0.1391;-0.0107] | 0.0180 | -0.0838 | [-0.1456;-0.0228] | 0.0040 | 0.1594 | [-0.0290;0.6047] | 0.0940 |

D ACME = Average Causal Mediation Effect

ADE = Average Direct Effect
