# Supplementary material for: From blood to lung tissue: effect of cigarette smoke on DNA methylation and lung function
Source: Respir Res. 2018 Nov 3;19:212. doi: 10.1186/s12931-018-0904-y (PMC6215675; doi:10.1186/s12931-018-0904-y)
Supplement: Supplementary file 3 — Subject characteristics lung tissue cohort. (DOCX 17 kb) [file 12931_2018_904_MOESM3_ESM.docx]

*Additional file 3: Subject characteristics lung tissue cohort*

|  | **Never smokers** | **Ex-smokers** | **Current smokers** |
| --- | --- | --- | --- |
| **Number** | 16 | 15 | 16 |
| **Males**, N (%) | 5 (31.3) | 11 (73.3) | 9 (56.3) |
| **Age**, years (range) | 57 (26 - 82) | 68 (56 - 82) | 56 (42 - 71) |
|  |  |  |  |
| **Pack years**, years, mean (range) | - | 39 (10 - 60) | 33 (8 - 81) |
| **Years cessation** (range) | - | 11 (1 – 25) | - |
|  |  |  |  |
| **FEV_1_/FVC**, (%), mean (range) | 75.3 (45.0-89.9) | 58.8 (43.5-79.4) | 67.2 (43.8-79.5) |
| **FEV_1_**,(%pred), mean (range) | 85.6 (23.0-121.0)* | 64.4 (30.7-97.9) † | 74.5 (44.9-101.4) ‡ |
| **COPD cases**, N (%) | 3 (18.8) | 14 (93.3) | 9 (56.3) |
| **GOLD COPD Stage ≥ 2**, N (%) | 2 (14.3)* | 11 (84.6) † | 4 (36.4) ‡ |

* Only data of 14 subjects was available

† Only data of 13 subjects was available

‡ Only data of 11 subjects was available
