# Supplementary material for: From blood to lung tissue: effect of cigarette smoke on DNA methylation and lung function
Source: Respir Res. 2018 Nov 3;19:212. doi: 10.1186/s12931-018-0904-y (PMC6215675; doi:10.1186/s12931-018-0904-y)
Supplement: Supplementary file 4 — Overview results of the association between DNA methylation and smoking status in lung tissue. (DOCX 19 kb) [file 12931_2018_904_MOESM4_ESM.docx]

*Additional file 4: Overview results of the association between DNA methylation and smoking status in lung tissue*

|  |  | Never vs Current smoking | | | Never vs Ex-smoking | | | Ex- vs Current smoking | | |
| --- | --- | --- | --- | --- | --- | --- | --- | --- | --- | --- |
| CpG-site | **Gene** | **Beta** | **SE** | **P-value** | **Beta** | **SE** | **P-value** | **Beta** | **SE** | **P-value** |
| cg05577921 | AHRR | -14.892 | 2.138 | 3.299E-12 | -7.108 | 2.510 | 4.634E-03 | -7.784 | 2.415 | 1.269E-03 |
| cg21161138 | AHRR | -8.822 | 1.341 | 4.780E-11 | -4.138 | 1.579 | 8.764E-03 | -4.684 | 1.521 | 2.073E-03 |
| cg06126421 | 6p21.33 | -6.919 | 1.751 | 7.786E-05 | -3.507 | 2.056 | 0.088 | -3.412 | 1.978 | 0.085 |
| cg05951221 | 2q37.1 | -3.302 | 0.991 | 8.659E-04 | -1.994 | 1.164 | 0.087 | -1.309 | 1.120 | 0.243 |
| cg21566642 | 2q37.1 | -2.219 | 1.086 | 0.041 | 0.453 | 1.275 | 0.722 | -2.672 | 1.227 | 0.029 |
| cg09935388 | GFI1 | -3.400 | 1.907 | 0.075 | -2.778 | 2.185 | 0.204 | -0.622 | 2.139 | 0.771 |
| cg24859433 | 6p21.33 | -2.312 | 1.910 | 0.226 | -0.450 | 2.243 | 0.841 | -1.862 | 2.158 | 0.388 |
| cg03636183 | F2RL3 | -1.716 | 1.498 | 0.252 | -0.727 | 1.756 | 0.679 | -0.989 | 1.671 | 0.554 |
| cg22994830 | PRKAR1B | -1.311 | 1.713 | 0.444 | -0.481 | 2.011 | 0.811 | -0.830 | 1.935 | 0.668 |
