# Supplementary material for: From blood to lung tissue: effect of cigarette smoke on DNA methylation and lung function
Source: Respir Res. 2018 Nov 3;19:212. doi: 10.1186/s12931-018-0904-y (PMC6215675; doi:10.1186/s12931-018-0904-y)
Supplement: Supplementary file 5 — Overview results of the eQTM analysis. (PDF 553 kb) [file 12931_2018_904_MOESM5_ESM.pdf]

Additional file 5: Overview results eQTM analysis

CpG name	Beta	SE	P-value	Bonferroni	Chromosome	bp_meth	bp_Gene	Annotation CpG	Probe	Genename
cg05575921	-0.1039	0.0184	1.80E-08	9.18E-07	5	373378	357290	AHRR	100155403_TGI_at	AHRR
cg21161138	-0.1787	0.0348	2.74E-07	1.45E-05	5	399360	357290	AHRR	100155403_TGI_at	AHRR
cg05951221	0.0432	0.0096	6.66E-06	3.53E-04	2	233284402	233824955		100136522_TGI_at	ATG16L1
cg21566642	0.0420	0.0098	1.64E-05	8.71E-04	2	233284661	233824955		100136522_TGI_at	ATG16L1
cg05951221	-0.0469	0.0122	1.19E-04	6.29E-03	2	233284402	232534536		100140869_TGI_at	DIS3L2
cg06126421	-0.1529	0.0380	5.68E-05	7.27E-03	6	30720080	31059463		100159175_TGI_at	MUC21
cg21566642	-0.0417	0.0123	6.84E-04	3.63E-02	2	233284661	232534536		100140869_TGI_at	DIS3L2
cg06126421	-0.0188	0.0053	3.57E-04	4.58E-02	6	30720080	30796135		100300978_TGI_at	TUBB
cg06126421	-0.1176	0.0340	5.37E-04	6.87E-02	6	30720080	31059528		100309111_TGI_at	MUC21
cg05951221	0.0311	0.0097	1.31E-03	6.96E-02	2	233284402	232305390		100152901_TGI_at	PDE6D
cg05575921	-0.0137	0.0043	1.57E-03	8.01E-02	5	373378	1306286	AHRR	100144642_TGI_at	TERT
cg21566642	0.0304	0.0099	2.22E-03	1.18E-01	2	233284661	232305390		100152901_TGI_at	PDE6D
cg21161138	-0.0433	0.0144	2.56E-03	1.35E-01	5	399360	474045	AHRR	100309585_TGI_at	AHRR
cg05575921	-0.0102	0.0034	2.74E-03	1.40E-01	5	373378	654859	AHRR	100152132_TGI_at	
cg05575921	-0.0115	0.0039	3.03E-03	1.55E-01	5	373378	357298	AHRR	100162325_TGI_at	AHRR
cg06126421	-0.0254	0.0080	1.52E-03	1.94E-01	6	30720080	31691011		100151160_TGI_at	AIF1
cg05951221	-0.0239	0.0086	5.64E-03	2.99E-01	2	233284402	232959742		100124762_TGI_at	ECEL1P2
cg05575921	-0.0146	0.0053	6.23E-03	3.18E-01	5	373378	1278469	AHRR	100144282_TGI_at	SLC6A18
cg21566642	0.0435	0.0159	6.25E-03	3.31E-01	2	233284661	234147014		100145437_TGI_at	
cg05951221	-0.0314	0.0116	6.89E-03	3.65E-01	2	233284402	232534637		100309749_TGI_at	DIS3L2
cg05575921	0.0334	0.0126	7.94E-03	4.05E-01	5	373378	254500	AHRR	100142366_TGI_at	
cg21161138	-0.0283	0.0107	8.36E-03	4.43E-01	5	399360	357291	AHRR	100138821_TGI_at	AHRR
cg05951221	-0.0736	0.0284	9.63E-03	5.10E-01	2	233284402	233206567		100156680_TGI_at	EFHD1
cg05575921	0.0224	0.0087	1.01E-02	5.15E-01	5	373378	237421	AHRR	100126945_TGI_at	PLEKHG4B
cg21566642	-0.0303	0.0118	1.05E-02	5.58E-01	2	233284661	232534637		100309749_TGI_at	DIS3L2
cg05951221	0.0412	0.0161	1.06E-02	5.60E-01	2	233284402	234147014		100145437_TGI_at	
cg05575921	-0.0105	0.0042	1.17E-02	5.95E-01	5	373378	923403	AHRR	100310904_TGI_at	BRD9
cg06126421	-0.0256	0.0092	5.37E-03	6.87E-01	6	30720080	31623350		100152647_TGI_at	NFKBIL1
cg05575921	-0.0194	0.0079	1.41E-02	7.17E-01	5	373378	474045	AHRR	100309585_TGI_at	AHRR
cg05575921	-0.0124	0.0050	1.41E-02	7.22E-01	5	373378	495578	AHRR	100146413_TGI_at	

CpG name	Beta	SE	P-value	Bonferroni	Chromosome	bp_meth	bp_Gene	Annotation CpG	Probe	Genename
cg21566642	-0.0687	0.0279	1.37E-02	7.25E-01	2	233284661	233206567		100156680_TGI_at	EFHD1
cg05951221	-0.0269	0.0109	1.37E-02	7.29E-01	2	233284402	233029076		100157259_TGI_at	ALPI
cg06126421	-0.0189	0.0069	6.04E-03	7.73E-01	6	30720080	30050870		100132949_TGI_at	HCG9
cg06126421	0.0497	0.0182	6.46E-03	8.27E-01	6	30720080	29867662		100141210_TGI_at	
cg06126421	0.0398	0.0147	6.78E-03	8.68E-01	6	30720080	31538937		100144394_TGI_at	HCP5
cg05575921	-0.0157	0.0067	1.91E-02	9.74E-01	5	373378	357291	AHRR	100138821_TGI_at	AHRR
cg05575921	-0.0112	0.0049	2.18E-02	1	5	373378	1057830	AHRR	100162877_TGI_at	
cg05575921	-0.0095	0.0046	3.73E-02	1	5	373378	420579	AHRR	100132989_TGI_at	AHRR
cg05575921	-0.0120	0.0060	4.51E-02	1	5	373378	1163893	AHRR	100129229_TGI_at	
cg05575921	-0.0092	0.0048	5.49E-02	1	5	373378	248892	AHRR	100148989_TGI_at	
cg05575921	0.0333	0.0177	5.98E-02	1	5	373378	946003	AHRR	100151667_TGI_at	TRIP13
cg05575921	-0.0106	0.0057	6.03E-02	1	5	373378	496333	AHRR	100137677_TGI_at	EXOC3
cg05575921	-0.0168	0.0097	8.30E-02	1	5	373378	136266	AHRR	100158534_TGI_at	
cg05575921	-0.0076	0.0046	1.01E-01	1	5	373378	1032479	AHRR	100153219_TGI_at	
cg05575921	0.0235	0.0150	1.17E-01	1	5	373378	713890	AHRR	100313551_TGI_at	TPPP
cg05575921	-0.0079	0.0051	1.25E-01	1	5	373378	849287	AHRR	100307772_TGI_at	ZDHHC11
cg05575921	-0.0146	0.0100	1.44E-01	1	5	373378	532246	AHRR	100123914_TGI_at	
cg05575921	-0.0050	0.0035	1.49E-01	1	5	373378	271355	AHRR	100302759_TGI_at	SDHA
cg05575921	-0.0067	0.0047	1.55E-01	1	5	373378	525126	AHRR	100307857_TGI_at	
cg05575921	0.0114	0.0085	1.80E-01	1	5	373378	237421	AHRR	100306701_TGI_at	PLEKHG4B
cg05575921	-0.0116	0.0090	2.00E-01	1	5	373378	1277671	AHRR	100125947_TGI_at	SLC6A19
cg05575921	-0.0130	0.0106	2.21E-01	1	5	373378	1062167	AHRR	100154736_TGI_at	NKD2
cg05575921	0.0169	0.0140	2.28E-01	1	5	373378	713890	AHRR	100144166_TGI_at	TPPP
cg05575921	-0.0118	0.0102	2.46E-01	1	5	373378	944761	AHRR	100304823_TGI_at	BRD9
cg05575921	-0.0064	0.0056	2.53E-01	1	5	373378	327872	AHRR	100131641_TGI_at	PDCD6
cg05575921	-0.0066	0.0058	2.56E-01	1	5	373378	244625	AHRR	100123951_TGI_at	
cg05575921	-0.0116	0.0104	2.64E-01	1	5	373378	1062076	AHRR	100309999_TGI_at	NKD2
cg05575921	-0.0044	0.0042	2.91E-01	1	5	373378	1254765	AHRR	100140783_TGI_at	SLC6A19
cg05575921	0.0064	0.0063	3.11E-01	1	5	373378	1103488	AHRR	100126447_TGI_at	SLC12A7
cg05575921	-0.0046	0.0049	3.44E-01	1	5	373378	193372	AHRR	100138179_TGI_at	PLEKHG4B
cg05575921	0.0036	0.0039	3.55E-01	1	5	373378	513536	AHRR	100131916_TGI_at	
cg05575921	-0.0219	0.0251	3.84E-01	1	5	373378	1226255	AHRR	100311848_TGI_at	

CpG name	Beta	SE	P-value	Bonferroni	Chromosome	bp_meth	bp_Gene	Annotation CpG	Probe	Genename
cg05575921	-0.0037	0.0044	4.00E-01	1	5	373378	1254709	AHRR	100125143_TGI_at	SLC6A19
cg05575921	0.0168	0.0202	4.06E-01	1	5	373378	848721	AHRR	100133783_TGI_at	ZDHHC11
cg05575921	0.0149	0.0181	4.09E-01	1	5	373378	763474	AHRR	100300181_TGI_at	ZDHHC11
cg05575921	-0.0045	0.0056	4.24E-01	1	5	373378	193680	AHRR	100155610_TGI_at	PLEKHG4B
cg05575921	-0.0025	0.0036	4.82E-01	1	5	373378	526424	AHRR	100150375_TGI_at	SLC9A3
cg05575921	-0.0027	0.0041	5.02E-01	1	5	373378	665404	AHRR	100151246_TGI_at	CEP72
cg05575921	0.0017	0.0038	6.55E-01	1	5	373378	324777	AHRR	100305370_TGI_at	PDCD6
cg05575921	0.0016	0.0085	8.47E-01	1	5	373378	523626	AHRR	100304150_TGI_at	
cg05575921	0.0011	0.0072	8.77E-01	1	5	373378	523626	AHRR	100141396_TGI_at	
cg05575921	0.0006	0.0046	8.90E-01	1	5	373378	277519	AHRR	100304547_TGI_at	SDHA
cg05575921	0.0007	0.0064	9.14E-01	1	5	373378	327872	AHRR	100312117_TGI_at	PDCD6
cg05575921	-0.0009	0.0097	9.26E-01	1	5	373378	258073	AHRR	100311673_TGI_at	CCDC127
cg05575921	0.0010	0.0112	9.30E-01	1	5	373378	257874	AHRR	100127701_TGI_at	CCDC127
cg05575921	-0.0001	0.0047	9.78E-01	1	5	373378	1272703	AHRR	100124001_TGI_at	SLC6A19
cg05951221	-0.0302	0.0145	3.73E-02	1	2	233284402	232979795		100142619_TGI_at	ALPPL2
cg05951221	-0.0320	0.0167	5.52E-02	1	2	233284402	233443237		100148559_TGI_at	C2orf82
cg05951221	-0.0152	0.0083	6.69E-02	1	2	233284402	233448752		100133682_TGI_at	C2orf82
cg05951221	0.0333	0.0191	8.17E-02	1	2	233284402	233270258		100302206_TGI_at	GIGYF2
cg05951221	-0.0223	0.0131	8.92E-02	1	2	233284402	232990987		100146285_TGI_at	
cg05951221	-0.0311	0.0186	9.50E-02	1	2	233284402	233254953		100303862_TGI_at	EFHD1
cg05951221	0.0316	0.0204	1.22E-01	1	2	233284402	233742741		100123878_TGI_at	INPP5D
cg05951221	-0.0121	0.0085	1.56E-01	1	2	233284402	233033426		100130494_TGI_at	
cg05951221	0.0253	0.0180	1.61E-01	1	2	233284402	234058990		100135825_TGI_at	USP40
cg05951221	-0.0134	0.0101	1.85E-01	1	2	233284402	232925503		100157308_TGI_at	
cg05951221	0.0210	0.0163	1.99E-01	1	2	233284402	233633311		100157967_TGI_at	INPP5D
cg05951221	0.0181	0.0142	2.04E-01	1	2	233284402	233633311		100300545_TGI_at	INPP5D
cg05951221	-0.0123	0.0101	2.25E-01	1	2	233284402	233108610		100159750_TGI_at	CHRND
cg05951221	-0.0187	0.0160	2.44E-01	1	2	233284402	233451639		100138454_TGI_at	NGEF
cg05951221	-0.0123	0.0108	2.56E-01	1	2	233284402	233099165		100161687_TGI_at	CHRND
cg05951221	0.0094	0.0089	2.94E-01	1	2	233284402	233405810		100124019_TGI_at	GIGYF2
cg05951221	0.0290	0.0278	2.96E-01	1	2	233284402	232382799		100153985_TGI_at	
cg05951221	0.0147	0.0147	3.19E-01	1	2	233284402	233276374		100304412_TGI_at	GIGYF2

CpG name	Beta	SE	P-value	Bonferroni	Chromosome	bp_meth	bp_Gene	Annotation CpG	Probe	Genename
cg05951221	-0.0090	0.0092	3.30E-01	1	2	233284402	233585567		100128184_TGI_at	
cg05951221	-0.0117	0.0125	3.46E-01	1	2	233284402	233881206		100127704_TGI_at	SAG
cg05951221	-0.0086	0.0097	3.79E-01	1	2	233284402	233605625		100125390_TGI_at	NEU2
cg05951221	-0.0098	0.0114	3.93E-01	1	2	233284402	232498378		100144674_TGI_at	NPPC
cg05951221	-0.0162	0.0216	4.54E-01	1	2	233284402	233339103		100153794_TGI_at	KCNJ13
cg05951221	-0.0107	0.0160	5.05E-01	1	2	233284402	233633279		100308965_TGI_at	INPP5D
cg05951221	0.0069	0.0106	5.14E-01	1	2	233284402	232359405		100124382_TGI_at	COPS7B
cg05951221	0.0125	0.0203	5.38E-01	1	2	233284402	232534637		100162400_TGI_at	DIS3L2
cg05951221	-0.0061	0.0107	5.68E-01	1	2	233284402	233901986		100306256_TGI_at	SAG
cg05951221	-0.0101	0.0180	5.72E-01	1	2	233284402	233123601		100127022_TGI_at	EIF4E2
cg05951221	-0.0053	0.0096	5.77E-01	1	2	233284402	233123639		100308088_TGI_at	EIF4E2
cg05951221	0.0061	0.0115	5.99E-01	1	2	233284402	233112680		100161148_TGI_at	CHRNG
cg05951221	-0.0064	0.0128	6.15E-01	1	2	233284402	233123600		100302691_TGI_at	EIF4E2
cg05951221	-0.0245	0.0515	6.34E-01	1	2	233284402	232951591		100302996_TGI_at	ALPP
cg05951221	-0.0059	0.0148	6.89E-01	1	2	233284402	233139867		100305783_TGI_at	EIF4E2
cg05951221	-0.0039	0.0105	7.11E-01	1	2	233284402	234048910		100128277_TGI_at	
cg05951221	-0.0034	0.0113	7.61E-01	1	2	233284402	233123600		100129016_TGI_at	EIF4E2
cg05951221	0.0053	0.0183	7.71E-01	1	2	233284402	233270284		100142158_TGI_at	GIGYF2
cg05951221	0.0040	0.0160	8.01E-01	1	2	233284402	233633279		100149718_TGI_at	INPP5D
cg05951221	-0.0060	0.0263	8.20E-01	1	2	233284402	233121022		100134110_TGI_at	TIGD1
cg05951221	-0.0060	0.0311	8.46E-01	1	2	233284402	233121022		100301223_TGI_at	TIGD1
cg05951221	0.0022	0.0115	8.49E-01	1	2	233284402	233052780		100151962_TGI_at	ECEL1
cg05951221	0.0026	0.0162	8.75E-01	1	2	233284402	232359387		100312114_TGI_at	COPS7B
cg05951221	-0.0016	0.0104	8.81E-01	1	2	233284402	233093416		100142878_TGI_at	
cg05951221	0.0016	0.0123	8.93E-01	1	2	233284402	234048904		100302018_TGI_at	USP40
cg05951221	-0.0026	0.0264	9.21E-01	1	2	233284402	234041030		100134859_TGI_at	DGKD
cg05951221	0.0004	0.0170	9.80E-01	1	2	233284402	233927891		100131135_TGI_at	DGKD
cg06126421	-0.0170	0.0065	9.16E-03	1	6	30720080	31191331		100141380_TGI_at	CDSN
cg06126421	0.0493	0.0192	1.03E-02	1	6	30720080	31430492		100313880_TGI_at	
cg06126421	0.0312	0.0124	1.17E-02	1	6	30720080	30617145		100134466_TGI_at	
cg06126421	0.0331	0.0133	1.29E-02	1	6	30720080	31555731		100138417_TGI_at	
cg06126421	0.0273	0.0114	1.66E-02	1	6	30720080	29799095		100303129_TGI_at	HLA-F

CpG name	Beta	SE	P-value	Bonferroni	Chromosome	bp_meth	bp_Gene	Annotation CpG	Probe	Genename
cg06126421	0.0285	0.0122	1.91E-02	1	6	30720080	29902734		100160221_TGI_at	HLA-G
cg06126421	-0.0151	0.0065	2.05E-02	1	6	30720080	30693584		100155044_TGI_at	MRPS18B
cg06126421	0.0200	0.0086	2.06E-02	1	6	30720080	29903802		100138030_TGI_at	HLA-G
cg06126421	0.0196	0.0087	2.38E-02	1	6	30720080	30565499		100306223_TGI_at	HLA-E
cg06126421	0.0370	0.0165	2.53E-02	1	6	30720080	29799189		100311606_TGI_at	HLA-F
cg06126421	-0.0124	0.0056	2.64E-02	1	6	30720080	30142910		100139766_TGI_at	PPP1R11
cg06126421	-0.0425	0.0193	2.74E-02	1	6	30720080	30146022		100135776_TGI_at	RNF39
cg06126421	0.0295	0.0140	3.49E-02	1	6	30720080	31273577		100300925_TGI_at	HCG27
cg06126421	-0.0162	0.0077	3.49E-02	1	6	30720080	30145332		100132397_TGI_at	
cg06126421	-0.0167	0.0081	3.88E-02	1	6	30720080	30032417		100300018_TGI_at	
cg06126421	0.0295	0.0150	4.90E-02	1	6	30720080	31546990		100303796_TGI_at	
cg06126421	-0.0178	0.0091	5.01E-02	1	6	30720080	30763805		100125579_TGI_at	NRM
cg06126421	-0.0242	0.0124	5.16E-02	1	6	30720080	30973994		100305113_TGI_at	DDR1
cg06126421	-0.0277	0.0143	5.17E-02	1	6	30720080	31007105		100125489_TGI_at	SFTA2
cg06126421	0.0484	0.0253	5.58E-02	1	6	30720080	31249491		100304000_TGI_at	PSORS1C3
cg06126421	-0.0195	0.0102	5.64E-02	1	6	30720080	30959839		100161807_TGI_at	DDR1
cg06126421	0.0384	0.0202	5.68E-02	1	6	30720080	29867662		100304197_TGI_at	
cg06126421	-0.0376	0.0207	6.96E-02	1	6	30720080	29847069		100309580_TGI_at	
cg06126421	-0.0191	0.0112	8.86E-02	1	6	30720080	31661949		100149313_TGI_at	LST1
cg06126421	0.0263	0.0158	9.64E-02	1	6	30720080	31664650		100125842_TGI_at	NCR3
cg06126421	-0.0118	0.0072	9.97E-02	1	6	30720080	30983955		100147044_TGI_at	GTF2H4
cg06126421	0.0350	0.0213	9.98E-02	1	6	30720080	30279550		100153401_TGI_at	TRIM26
cg06126421	0.0120	0.0079	1.27E-01	1	6	30720080	29810784		100141578_TGI_at	RP3-377H14.5
cg06126421	-0.0176	0.0119	1.38E-01	1	6	30720080	31218194		100126937_TGI_at	CCHCR1
cg06126421	-0.0138	0.0097	1.57E-01	1	6	30720080	30647148		100313354_TGI_at	ABCF1
cg06126421	0.0268	0.0190	1.60E-01	1	6	30720080	29802358		100311825_TGI_at	RP3-377H14.5
cg06126421	0.0271	0.0208	1.92E-01	1	6	30720080	31273577		100128894_TGI_at	HCG27
cg06126421	-0.0123	0.0095	1.94E-01	1	6	30720080	31573944		100161520_TGI_at	MICB
cg06126421	-0.0335	0.0265	2.07E-01	1	6	30720080	30233436		100135516_TGI_at	TRIM10
cg06126421	-0.0144	0.0114	2.07E-01	1	6	30720080	31475540		100304001_TGI_at	
cg06126421	-0.0118	0.0095	2.13E-01	1	6	30720080	31711719		100307012_TGI_at	BAT2
cg06126421	-0.0118	0.0095	2.13E-01	1	6	30720080	31696428		100301381_TGI_at	BAT2

CpG name	Beta	SE	P-value	Bonferroni	Chromosome	bp_meth	bp_Gene	Annotation CpG	Probe	Genename
cg06126421	0.0040	0.0033	2.20E-01	1	6	30720080	31605976		100162740_TGI_at	BAT1
cg06126421	-0.0131	0.0107	2.21E-01	1	6	30720080	31487031		100305324_TGI_at	
cg06126421	-0.0225	0.0184	2.21E-01	1	6	30720080	31470045		100155331_TGI_at	
cg06126421	-0.0080	0.0066	2.26E-01	1	6	30720080	30776070		100155243_TGI_at	MDC1
cg06126421	-0.0109	0.0091	2.31E-01	1	6	30720080	30647148		100124255_TGI_at	ABCF1
cg06126421	0.0057	0.0048	2.36E-01	1	6	30720080	31606189		100306979_TGI_at	BAT1
cg06126421	-0.0062	0.0053	2.43E-01	1	6	30720080	30728885		100131277_TGI_at	DHX16
cg06126421	-0.0116	0.0101	2.51E-01	1	6	30720080	31356094		100139476_TGI_at	
cg06126421	-0.0153	0.0137	2.62E-01	1	6	30720080	30800255		100307304_TGI_at	FLOT1
cg06126421	-0.0096	0.0088	2.74E-01	1	6	30720080	31186980		100155167_TGI_at	C6orf15
cg06126421	-0.0191	0.0175	2.75E-01	1	6	30720080	31270960		100311757_TGI_at	
cg06126421	-0.0125	0.0115	2.78E-01	1	6	30720080	30817375		100313972_TGI_at	FLOT1
cg06126421	-0.0098	0.0091	2.82E-01	1	6	30720080	30803491		100156505_TGI_at	FLOT1
cg06126421	-0.0203	0.0189	2.84E-01	1	6	30720080	31620220		100142732_TGI_at	ATP6V1G2
cg06126421	-0.0118	0.0112	2.92E-01	1	6	30720080	30137014		100158228_TGI_at	ZNRD1
cg06126421	-0.0060	0.0058	2.98E-01	1	6	30720080	30465971		100152513_TGI_at	
cg06126421	-0.0103	0.0100	3.02E-01	1	6	30720080	30815953		100308169_TGI_at	FLOT1
cg06126421	0.0059	0.0057	3.03E-01	1	6	30720080	31344828		100313121_TGI_at	HLA-C
cg06126421	-0.0133	0.0129	3.05E-01	1	6	30720080	30817013		100304636_TGI_at	FLOT1
cg06126421	0.0057	0.0057	3.18E-01	1	6	30720080	31429875		100300038_TGI_at	HLA-B
cg06126421	-0.0102	0.0112	3.64E-01	1	6	30720080	30989960		100151371_TGI_at	VARS2
cg06126421	-0.0092	0.0106	3.89E-01	1	6	30720080	31604717		100147446_TGI_at	MCCD1
cg06126421	0.0071	0.0085	4.01E-01	1	6	30720080	30723176		100153402_TGI_at	C6orf136
cg06126421	-0.0056	0.0068	4.05E-01	1	6	30720080	30239440		100149405_TGI_at	TRIM15
cg06126421	0.0077	0.0093	4.11E-01	1	6	30720080	31344508		100302942_TGI_at	HLA-C
cg06126421	-0.0085	0.0108	4.35E-01	1	6	30720080	31698835		100155673_TGI_at	SNORA38
cg06126421	-0.0333	0.0429	4.37E-01	1	6	30720080	29748256		100303994_TGI_at	ZFP57
cg06126421	-0.0074	0.0097	4.43E-01	1	6	30720080	30260212		100126192_TGI_at	TRIM26
cg06126421	-0.0069	0.0092	4.56E-01	1	6	30720080	31696575		100302712_TGI_at	BAT2
cg06126421	-0.0072	0.0099	4.63E-01	1	6	30720080	31696428		100139818_TGI_at	BAT2
cg06126421	0.0187	0.0256	4.66E-01	1	6	30720080	30082343		100136412_TGI_at	HLA-G
cg06126421	-0.0126	0.0177	4.77E-01	1	6	30720080	30676161		100159380_TGI_at	PPP1R10

CpG name	Beta	SE	P-value	Bonferroni	Chromosome	bp_meth	bp_Gene	Annotation CpG	Probe	Genename
cg06126421	-0.0222	0.0315	4.80E-01	1	6	30720080	30818954		100135011_TGI_at	IER3
cg06126421	0.0092	0.0132	4.87E-01	1	6	30720080	30081147		100125007_TGI_at	C6orf12
cg06126421	0.0058	0.0083	4.88E-01	1	6	30720080	31344506		100309484_TGI_at	HLA-C
cg06126421	0.0049	0.0072	4.93E-01	1	6	30720080	29735073		100300527_TGI_at	MOG
cg06126421	-0.0158	0.0234	4.98E-01	1	6	30720080	31190601		100121628_TGI_at	PSORS1C1
cg06126421	-0.0065	0.0097	5.02E-01	1	6	30720080	30621674		100149228_TGI_at	GNL1
cg06126421	-0.0049	0.0074	5.14E-01	1	6	30720080	30226727		100140268_TGI_at	
cg06126421	0.0113	0.0175	5.18E-01	1	6	30720080	30000478		100123483_TGI_at	HCG4P6
cg06126421	0.0031	0.0049	5.18E-01	1	6	30720080	30018309		100129349_TGI_at	HLA-A
cg06126421	-0.0051	0.0079	5.20E-01	1	6	30720080	29732787		100137900_TGI_at	MOG
cg06126421	0.0053	0.0087	5.48E-01	1	6	30720080	30178654		100155181_TGI_at	TRIM31
cg06126421	-0.0055	0.0099	5.79E-01	1	6	30720080	29826321		100304754_TGI_at	
cg06126421	0.0061	0.0113	5.87E-01	1	6	30720080	30367388		100308127_TGI_at	HCG18
cg06126421	0.0047	0.0088	5.93E-01	1	6	30720080	30147130		100312608_TGI_at	RNF39
cg06126421	-0.0032	0.0064	6.15E-01	1	6	30720080	31213289		100124666_TGI_at	PSORS1C2
cg06126421	-0.0032	0.0064	6.16E-01	1	6	30720080	31691277		100140033_TGI_at	AIF1
cg06126421	0.0081	0.0164	6.21E-01	1	6	30720080	31648071		100162143_TGI_at	LTA
cg06126421	-0.0057	0.0116	6.26E-01	1	6	30720080	30366765		100131217_TGI_at	HCG18
cg06126421	0.0034	0.0075	6.56E-01	1	6	30720080	31190505		100306170_TGI_at	PSORS1C1
cg06126421	-0.0087	0.0199	6.64E-01	1	6	30720080	31027537		100156384_TGI_at	DPCR1
cg06126421	-0.0044	0.0113	6.94E-01	1	6	30720080	31234303		100142285_TGI_at	TCF19
cg06126421	0.0041	0.0131	7.55E-01	1	6	30720080	30376549		100128261_TGI_at	
cg06126421	0.0058	0.0189	7.61E-01	1	6	30720080	30087854		100138253_TGI_at	HCG8
cg06126421	0.0039	0.0129	7.64E-01	1	6	30720080	30367130		100139688_TGI_at	HCG18
cg06126421	0.0021	0.0076	7.81E-01	1	6	30720080	31129962		100129279_TGI_at	HCG22
cg06126421	-0.0018	0.0067	7.84E-01	1	6	30720080	30702641		100126300_TGI_at	C6orf134
cg06126421	-0.0041	0.0149	7.85E-01	1	6	30720080	30227705		100133405_TGI_at	TRIM10
cg06126421	-0.0030	0.0112	7.93E-01	1	6	30720080	30752144		100158540_TGI_at	KIAA1949
cg06126421	0.0015	0.0057	7.99E-01	1	6	30720080	30775562		100302297_TGI_at	MDC1
cg06126421	-0.0054	0.0212	7.99E-01	1	6	30720080	31190848		100303039_TGI_at	CDSN
cg06126421	0.0017	0.0069	8.05E-01	1	6	30720080	30260576		100158973_TGI_at	
cg06126421	0.0051	0.0229	8.23E-01	1	6	30720080	29950912		100310523_TGI_at	

CpG name	Beta	SE	P-value	Bonferroni	Chromosome	bp_meth	bp_Gene	Annotation CpG	Probe	Genename
cg06126421	0.0021	0.0105	8.42E-01	1	6	30720080	31695126		100162932_TGI_at	
cg06126421	0.0042	0.0216	8.44E-01	1	6	30720080	31476834		100305045_TGI_at	
cg06126421	-0.0040	0.0216	8.55E-01	1	6	30720080	31656316		100154387_TGI_at	LTB
cg06126421	0.0012	0.0069	8.59E-01	1	6	30720080	30772681		100144058_TGI_at	
cg06126421	-0.0049	0.0304	8.71E-01	1	6	30720080	29974792		100143227_TGI_at	HCG2P7
cg06126421	-0.0015	0.0093	8.72E-01	1	6	30720080	30702631		100142981_TGI_at	C6orf134
cg06126421	0.0034	0.0232	8.84E-01	1	6	30720080	31476834		100162706_TGI_at	
cg06126421	0.0038	0.0262	8.84E-01	1	6	30720080	31651328		100145785_TGI_at	TNF
cg06126421	0.0009	0.0061	8.87E-01	1	6	30720080	30403018		100127148_TGI_at	TRIM39
cg06126421	-0.0014	0.0102	8.88E-01	1	6	30720080	30200931		100159888_TGI_at	
cg06126421	-0.0010	0.0073	8.90E-01	1	6	30720080	31620936		100308499_TGI_at	ATP6V1G2
cg06126421	0.0016	0.0123	8.94E-01	1	6	30720080	30076768		100145634_TGI_at	C6orf12
cg06126421	-0.0033	0.0260	9.00E-01	1	6	30720080	29974792		100300348_TGI_at	HCG2P7
cg06126421	0.0006	0.0053	9.04E-01	1	6	30720080	30227702		100161677_TGI_at	TRIM10
cg06126421	-0.0010	0.0129	9.41E-01	1	6	30720080	31138975		100123551_TGI_at	
cg06126421	0.0008	0.0110	9.41E-01	1	6	30720080	30147130		100142468_TGI_at	RNF39
cg06126421	-0.0009	0.0140	9.48E-01	1	6	30720080	30019070		100303756_TGI_at	HLA-A
cg06126421	0.0002	0.0056	9.74E-01	1	6	30720080	30562121		100142450_TGI_at	
cg06126421	-0.0002	0.0133	9.89E-01	1	6	30720080	30335352		100137319_TGI_at	
cg21161138	-0.0197	0.0099	4.62E-02	1	5	399360	1278469	AHRR	100144282_TGI_at	SLC6A18
cg21161138	-0.0133	0.0067	4.93E-02	1	5	399360	654859	AHRR	100152132_TGI_at	
cg21161138	-0.0126	0.0069	6.97E-02	1	5	399360	1272703	AHRR	100124001_TGI_at	SLC6A19
cg21161138	-0.0138	0.0078	7.83E-02	1	5	399360	1306286	AHRR	100144642_TGI_at	TERT
cg21161138	-0.0617	0.0410	1.32E-01	1	5	399360	1226255	AHRR	100311848_TGI_at	
cg21161138	0.0263	0.0175	1.33E-01	1	5	399360	257874	AHRR	100127701_TGI_at	CCDC127
cg21161138	0.0237	0.0167	1.57E-01	1	5	399360	258073	AHRR	100311673_TGI_at	CCDC127
cg21161138	-0.0122	0.0088	1.64E-01	1	5	399360	193372	AHRR	100138179_TGI_at	PLEKHG4B
cg21161138	0.0429	0.0327	1.89E-01	1	5	399360	763474	AHRR	100300181_TGI_at	ZDHHC11
cg21161138	-0.0111	0.0087	2.03E-01	1	5	399360	1057830	AHRR	100162877_TGI_at	
cg21161138	-0.0113	0.0089	2.04E-01	1	5	399360	248892	AHRR	100148989_TGI_at	
cg21161138	0.0373	0.0296	2.08E-01	1	5	399360	946003	AHRR	100151667_TGI_at	TRIP13
cg21161138	-0.0094	0.0075	2.12E-01	1	5	399360	277519	AHRR	100304547_TGI_at	SDHA

CpG name	Beta	SE	P-value	Bonferroni	Chromosome	bp_meth	bp_Gene	Annotation CpG	Probe	Genename
cg21161138	-0.0113	0.0093	2.28E-01	1	5	399360	193680	AHRR	100155610_TGI_at	PLEKHG4B
cg21161138	-0.0094	0.0082	2.53E-01	1	5	399360	357298	AHRR	100162325_TGI_at	AHRR
cg21161138	-0.0095	0.0083	2.56E-01	1	5	399360	923403	AHRR	100310904_TGI_at	BRD9
cg21161138	-0.0102	0.0095	2.79E-01	1	5	399360	420579	AHRR	100132989_TGI_at	AHRR
cg21161138	-0.0107	0.0103	2.98E-01	1	5	399360	495578	AHRR	100146413_TGI_at	
cg21161138	-0.0187	0.0186	3.14E-01	1	5	399360	944761	AHRR	100304823_TGI_at	BRD9
cg21161138	0.0340	0.0351	3.32E-01	1	5	399360	848721	AHRR	100133783_TGI_at	ZDHHC11
cg21161138	0.0097	0.0103	3.48E-01	1	5	399360	1103488	AHRR	100126447_TGI_at	SLC12A7
cg21161138	0.0095	0.0104	3.56E-01	1	5	399360	327872	AHRR	100312117_TGI_at	PDCD6
cg21161138	-0.0092	0.0102	3.66E-01	1	5	399360	244625	AHRR	100123951_TGI_at	
cg21161138	0.0239	0.0268	3.73E-01	1	5	399360	713890	AHRR	100313551_TGI_at	TPPP
cg21161138	0.0131	0.0166	4.29E-01	1	5	399360	237421	AHRR	100126945_TGI_at	PLEKHG4B
cg21161138	0.0114	0.0151	4.47E-01	1	5	399360	523626	AHRR	100304150_TGI_at	
cg21161138	-0.0131	0.0179	4.65E-01	1	5	399360	1062167	AHRR	100154736_TGI_at	NKD2
cg21161138	0.0170	0.0239	4.78E-01	1	5	399360	713890	AHRR	100144166_TGI_at	TPPP
cg21161138	-0.0055	0.0079	4.84E-01	1	5	399360	1370867	AHRR	100309906_TGI_at	CLPTM1L
cg21161138	0.0045	0.0067	5.02E-01	1	5	399360	513536	AHRR	100131916_TGI_at	
cg21161138	-0.0045	0.0068	5.03E-01	1	5	399360	324777	AHRR	100305370_TGI_at	PDCD6
cg21161138	-0.0071	0.0108	5.10E-01	1	5	399360	1163893	AHRR	100129229_TGI_at	
cg21161138	-0.0109	0.0167	5.14E-01	1	5	399360	136266	AHRR	100158534_TGI_at	
cg21161138	0.0094	0.0153	5.37E-01	1	5	399360	237421	AHRR	100306701_TGI_at	PLEKHG4B
cg21161138	-0.0106	0.0174	5.42E-01	1	5	399360	1062076	AHRR	100309999_TGI_at	NKD2
cg21161138	-0.0057	0.0098	5.62E-01	1	5	399360	327872	AHRR	100131641_TGI_at	PDCD6
cg21161138	-0.0051	0.0088	5.63E-01	1	5	399360	1370999	AHRR	100133970_TGI_at	CLPTM1L
cg21161138	-0.0037	0.0066	5.74E-01	1	5	399360	526424	AHRR	100150375_TGI_at	SLC9A3
cg21161138	-0.0034	0.0072	6.38E-01	1	5	399360	271355	AHRR	100302759_TGI_at	SDHA
cg21161138	-0.0088	0.0192	6.46E-01	1	5	399360	532246	AHRR	100123914_TGI_at	
cg21161138	-0.0031	0.0080	6.98E-01	1	5	399360	1032479	AHRR	100153219_TGI_at	
cg21161138	0.0044	0.0124	7.25E-01	1	5	399360	523626	AHRR	100141396_TGI_at	
cg21161138	0.0026	0.0075	7.26E-01	1	5	399360	665404	AHRR	100151246_TGI_at	CEP72
cg21161138	0.0068	0.0226	7.64E-01	1	5	399360	254500	AHRR	100142366_TGI_at	
cg21161138	0.0023	0.0085	7.87E-01	1	5	399360	525126	AHRR	100307857_TGI_at	

CpG name	Beta	SE	P-value	Bonferroni	Chromosome	bp_meth	bp_Gene	Annotation CpG	Probe	Genename
cg21161138	0.0024	0.0096	8.05E-01	1	5	399360	849287	AHRR	100307772_TGI_at	ZDHHC11
cg21161138	-0.0028	0.0160	8.62E-01	1	5	399360	1277671	AHRR	100125947_TGI_at	SLC6A19
cg21161138	-0.0011	0.0069	8.74E-01	1	5	399360	1254765	AHRR	100140783_TGI_at	SLC6A19
cg21161138	-0.0010	0.0076	8.95E-01	1	5	399360	1254709	AHRR	100125143_TGI_at	SLC6A19
cg21161138	-0.0006	0.0091	9.48E-01	1	5	399360	496333	AHRR	100137677_TGI_at	EXOC3
cg21566642	-0.0328	0.0149	2.81E-02	1	2	233284661	233443237		100148559_TGI_at	C2orf82
cg21566642	-0.0184	0.0086	3.21E-02	1	2	233284661	232959742		100124762_TGI_at	ECEL1P2
cg21566642	0.0369	0.0193	5.66E-02	1	2	233284661	233742741		100123878_TGI_at	INPP5D
cg21566642	0.0149	0.0079	6.02E-02	1	2	233284661	233405810		100124019_TGI_at	GIGYF2
cg21566642	0.0286	0.0177	1.07E-01	1	2	233284661	233633311		100157967_TGI_at	INPP5D
cg21566642	-0.0129	0.0083	1.20E-01	1	2	233284661	233448752		100133682_TGI_at	C2orf82
cg21566642	-0.0202	0.0133	1.28E-01	1	2	233284661	232990987		100146285_TGI_at	
cg21566642	-0.0165	0.0113	1.43E-01	1	2	233284661	233029076		100157259_TGI_at	ALPI
cg21566642	0.0169	0.0117	1.49E-01	1	2	233284661	233112680		100161148_TGI_at	CHRNG
cg21566642	-0.0204	0.0153	1.82E-01	1	2	233284661	232979795		100142619_TGI_at	ALPPL2
cg21566642	-0.0253	0.0190	1.83E-01	1	2	233284661	233254953		100303862_TGI_at	EFHD1
cg21566642	-0.0132	0.0100	1.84E-01	1	2	233284661	232925503		100157308_TGI_at	
cg21566642	-0.0130	0.0100	1.93E-01	1	2	233284661	233108610		100159750_TGI_at	CHRND
cg21566642	-0.0094	0.0082	2.55E-01	1	2	233284661	233033426		100130494_TGI_at	
cg21566642	-0.0195	0.0173	2.59E-01	1	2	233284661	233123601		100127022_TGI_at	EIF4E2
cg21566642	0.0161	0.0155	2.98E-01	1	2	233284661	234058990		100135825_TGI_at	USP40
cg21566642	0.0202	0.0197	3.05E-01	1	2	233284661	233270258		100302206_TGI_at	GIGYF2
cg21566642	0.0161	0.0165	3.28E-01	1	2	233284661	233276374		100304412_TGI_at	GIGYF2
cg21566642	0.0145	0.0149	3.32E-01	1	2	233284661	233633279		100149718_TGI_at	INPP5D
cg21566642	-0.0090	0.0095	3.41E-01	1	2	233284661	233605625		100125390_TGI_at	NEU2
cg21566642	-0.0148	0.0157	3.46E-01	1	2	233284661	232359387		100312114_TGI_at	COPS7B
cg21566642	0.0138	0.0149	3.57E-01	1	2	233284661	233633311		100300545_TGI_at	INPP5D
cg21566642	-0.0144	0.0158	3.62E-01	1	2	233284661	233451639		100138454_TGI_at	NGEF
cg21566642	-0.0449	0.0498	3.67E-01	1	2	233284661	232951591		100302996_TGI_at	ALPP
cg21566642	-0.0096	0.0123	4.35E-01	1	2	233284661	233881206		100127704_TGI_at	SAG
cg21566642	-0.0088	0.0115	4.45E-01	1	2	233284661	233099165		100161687_TGI_at	CHRND
cg21566642	0.0126	0.0177	4.79E-01	1	2	233284661	233270284		100142158_TGI_at	GIGYF2

CpG name	Beta	SE	P-value	Bonferroni	Chromosome	bp_meth	bp_Gene	Annotation CpG	Probe	Genename
cg21566642	-0.0078	0.0114	4.95E-01	1	2	233284661	233052780		100151962_TGI_at	ECEL1
cg21566642	0.0130	0.0197	5.09E-01	1	2	233284661	232534637		100162400_TGI_at	DIS3L2
cg21566642	0.0141	0.0303	6.43E-01	1	2	233284661	232382799		100153985_TGI_at	
cg21566642	-0.0045	0.0109	6.83E-01	1	2	233284661	232498378		100144674_TGI_at	NPPC
cg21566642	-0.0047	0.0121	7.01E-01	1	2	233284661	234048904		100302018_TGI_at	USP40
cg21566642	-0.0073	0.0214	7.32E-01	1	2	233284661	233339103		100153794_TGI_at	KCNJ13
cg21566642	-0.0091	0.0308	7.68E-01	1	2	233284661	233121022		100301223_TGI_at	TIGD1
cg21566642	-0.0027	0.0108	8.02E-01	1	2	233284661	233901986		100306256_TGI_at	SAG
cg21566642	0.0025	0.0106	8.11E-01	1	2	233284661	232359405		100124382_TGI_at	COPS7B
cg21566642	0.0032	0.0165	8.44E-01	1	2	233284661	233927891		100131135_TGI_at	DGKD
cg21566642	0.0021	0.0113	8.53E-01	1	2	233284661	233123600		100129016_TGI_at	EIF4E2
cg21566642	0.0017	0.0093	8.56E-01	1	2	233284661	233123639		100308088_TGI_at	EIF4E2
cg21566642	-0.0037	0.0263	8.89E-01	1	2	233284661	233121022		100134110_TGI_at	TIGD1
cg21566642	0.0016	0.0140	9.10E-01	1	2	233284661	233139867		100305783_TGI_at	EIF4E2
cg21566642	-0.0012	0.0107	9.12E-01	1	2	233284661	234048910		100128277_TGI_at	
cg21566642	0.0011	0.0163	9.45E-01	1	2	233284661	233633279		100308965_TGI_at	INPP5D
cg21566642	-0.0007	0.0102	9.47E-01	1	2	233284661	233093416		100142878_TGI_at	
cg21566642	-0.0007	0.0122	9.52E-01	1	2	233284661	233123600		100302691_TGI_at	EIF4E2
cg21566642	0.0004	0.0092	9.69E-01	1	2	233284661	233585567		100128184_TGI_at	
cg21566642	0.0008	0.0261	9.76E-01	1	2	233284661	234041030		100134859_TGI_at	DGKD
